# Modeling Dynamics of Cell-to-Cell Variability in TRAIL-Induced Apoptosis Explains Fractional Killing and Predicts Reversible Resistance

**DOI:** 10.1371/journal.pcbi.1003893

**Published:** 2014-10-23

**Authors:** François Bertaux, Szymon Stoma, Dirk Drasdo, Gregory Batt

**Affiliations:** 1 INRIA Paris-Rocquencourt, Le Chesnay, France; 2 Laboratoire Jacques-Louis Lions (LJLL), University of Paris 6 (UPMC) - CNRS (UMR7598), Paris, France; University of Virginia, United States of America

## Abstract

Isogenic cells sensing identical external signals can take markedly different decisions. Such decisions often correlate with pre-existing cell-to-cell differences in protein levels. When not neglected in signal transduction models, these differences are accounted for in a static manner, by assuming randomly distributed initial protein levels. However, this approach ignores the *a priori* non-trivial interplay between signal transduction and the source of this cell-to-cell variability: temporal fluctuations of protein levels in individual cells, driven by noisy synthesis and degradation. Thus, modeling protein fluctuations, rather than their consequences on the initial population heterogeneity, would set the quantitative analysis of signal transduction on firmer grounds. Adopting this dynamical view on cell-to-cell differences amounts to recast extrinsic variability into intrinsic noise. Here, we propose a generic approach to merge, in a systematic and principled manner, signal transduction models with stochastic protein turnover models. When applied to an established kinetic model of TRAIL-induced apoptosis, our approach markedly increased model prediction capabilities. One obtains a mechanistic explanation of yet-unexplained observations on fractional killing and non-trivial robust predictions of the temporal evolution of cell resistance to TRAIL in HeLa cells. Our results provide an alternative explanation to survival via induction of survival pathways since no TRAIL-induced regulations are needed and suggest that short-lived anti-apoptotic protein Mcl1 exhibit large and rare fluctuations. More generally, our results highlight the importance of accounting for stochastic protein turnover to quantitatively understand signal transduction over extended durations, and imply that fluctuations of short-lived proteins deserve particular attention.

## Author Summary

TRAIL induces apoptosis selectively in cancer cells and is currently tested in clinics. Having a mechanistic understanding of TRAIL resistance could help to limit its apparition. Several observations suggested that protein level fluctuations play an important role in TRAIL resistance and its acquisition. However, quantitative, systems-level approaches to investigate their role in cellular decision-making processes are lacking. We propose a generic and principled approach to extend signal transduction models with protein fluctuation models for all proteins in the pathway. The key aspect is to use standard protein fluctuation models for long-lived proteins. We show that its application to TRAIL-induced apoptosis provide a quantitative, mechanistic explanation to previously published but yet unexplained critical observations.

## Introduction

TNF-Related Apoptosis Inducing-Ligand (TRAIL) is a promising therapeutic agent against cancer because it induces apoptosis specifically in tumor cells [1]–[3]. This motivated dozens of clinical trials based on TRAIL-related therapies. However, efficiency was usually limited [4]. Most of the molecular events leading from TRAIL exposure to cell death are known [3]. After TRAIL binding to death receptors, initiator caspases are activated, which in turn promote effector caspases activation either directly or via a mitochondrial pathway (Fig. S1). In most cells, Mitochondrial Outer Membrane Permeabilization (MOMP) is required to efficiently activate effector caspases. Several kinetic models have been proposed to describe a part or all of those biochemical reactions [5]–[12].

Not all cells of an isogenic population die after TRAIL treatment, even at saturating ligand doses. This fractional killing property is widely shared among cell lines and is critical for therapeutical applications [13]. In addition, surviving cells were shown to be transiently resistant to a second TRAIL treatment. This reversible resistance property was observed in various cell lines and could also have important implications for therapy [14], [15]. Fractional killing is generally thought to result from cross talks between the apoptosis pathway and survival pathways [16]. Indeed, several studies reported that TRAIL increases the production of anti-apoptotic proteins, via the activation of survival pathways [17]–[19]. While fractional killing illustrates cell-to-cell variability in the decision between life and death, variability is also observed among cells that die: they commit to death after a highly variable delay from one another [13], [20]. This variability cannot be explained by differences in TRAIL-induced gene regulation: it is also observed when cells are co-treated with cycloheximide (CHX), an efficient inhibitor of protein synthesis [13]. Rather, it was proposed to originate mostly from pre-existing differences in the levels of proteins composing the apoptosis pathway. Indeed, recently divided sister cells die almost synchronously [13], [20], as expected if protein content is equally shared between daughters and if noise in signaling reactions play a marginal role. This explanation was supported by modeling: when taken as initial conditions of a deterministic, kinetic model describing signaling reactions, differences in protein levels are sufficient to explain observed variability in death times [13].

Another observation indicated that this cell-to-cell variability in protein levels is not frozen but result from a dynamical equilibrium driven by fluctuations in individual cells: death synchrony between sister cells was weaker as the duration between division and treatment increased [13], [20]. To quantitatively assess this effect, the previously mentioned modeling approach, where only the consequences of protein fluctuations (cell-to-cell variability at a fixed time) are accounted, is inadequate. Instead, protein fluctuations themselves should be modeled. The need to account for protein fluctuations is even more stringent when considering observations after treatment with TRAIL alone. In that case, protein synthesis is not blocked and thus can impact the decision between life and death. Even if TRAIL does not change protein synthesis (via induction of survival pathways), significant differences with TRAIL and CHX treatments are expected, as the constitutively noisy protein synthesis will interact with signaling reactions. Thus, before complexifying the model to account for eventual regulations via survival pathways induction, it is critical to assess how much can be explained when protein synthesis is not altered by TRAIL signaling. Here, we investigate this question by enabling a kinetic model of TRAIL-induced apoptosis with stochastic protein turnover for all proteins, following a generic and principled approach. To our knowledge, this is the first attempt to systematically include gene expression noise in a signal transduction model. It enriches the model with a fundamental property as the dynamics of cell-to-cell variability is represented, allowing disentangling the effects of constitutive protein fluctuations, signaling protein-protein reactions and potentially induced changes in protein synthesis.

## Results

### Extended vision of signal transduction pathways

Protein synthesis and degradation are subjected to noise, resulting in fluctuations of protein concentrations in individual cells and in cell-to-cell variability at the population level [21], [22]. Such variability could have consequences on signal transduction: aside of conventional epigenetic differences [23], unequal access to ligand molecules or simply noise in signaling reactions, it often contributes importantly to heterogeneous behavior within an isogenic population [13], [24]. One approach to account for those differences is to incorporate protein level variability as random initial conditions of an ODE model describing the signaling reactions (“extrinsic noise approach”) [13], [25]. However, variability is imposed at time zero and then behavior is deterministic: it is thus not appropriate to study transduction on long time scales, during which protein levels dynamically fluctuate [26]. A more natural manner to account for protein level variability is to represent their stochastic synthesis and degradation (“intrinsic noise approach”). Although several studies did account for cell-to-cell differences in protein levels in an extrinsic, static manner via random initial conditions [13], [27], [28], and many models of signal transduction considered the effect of noise in protein-protein reactions [29], [30] or in the expression of signal transduction target genes [31]–[33], no kinetic model of signal transduction pathways considering systematically noise in protein synthesis and degradation has been developed so far. Here by systematically we mean for all the proteins acting in the pathway.

**Figure 1:**
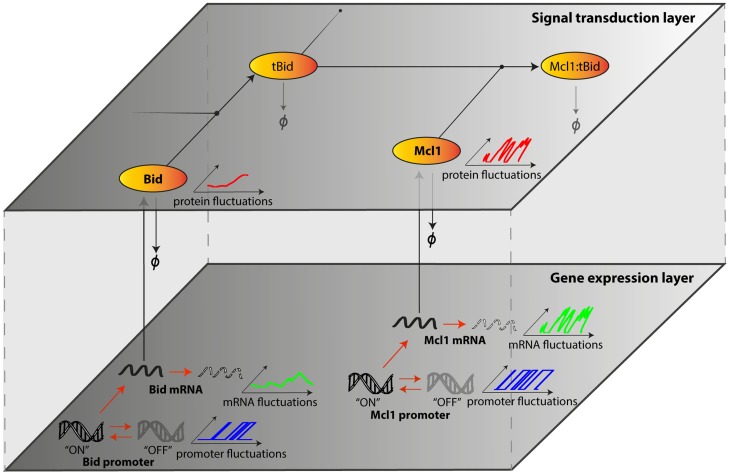
Accounting for stochastic protein turnover in signal transduction pathways. Scheme of the modeling approach. Protein-protein interactions mediating signal transduction (signal transduction layer) are modeled by ordinary differential equations. In parallel, promoter activity changes, mRNA production and degradation (gene expression layer) are seen as stochastic events and generate fluctuations in mRNA levels. This impacts the synthesis rates of the corresponding proteins. Together with protein degradation, it generates fluctuations in protein levels (here shown in absence of transduction). Only a fragment of the extrinsic apoptosis pathway is shown. Deterministic/stochastic interpretation of chemical reactions is represented with black/red arrows respectively.

**Figure 2:**
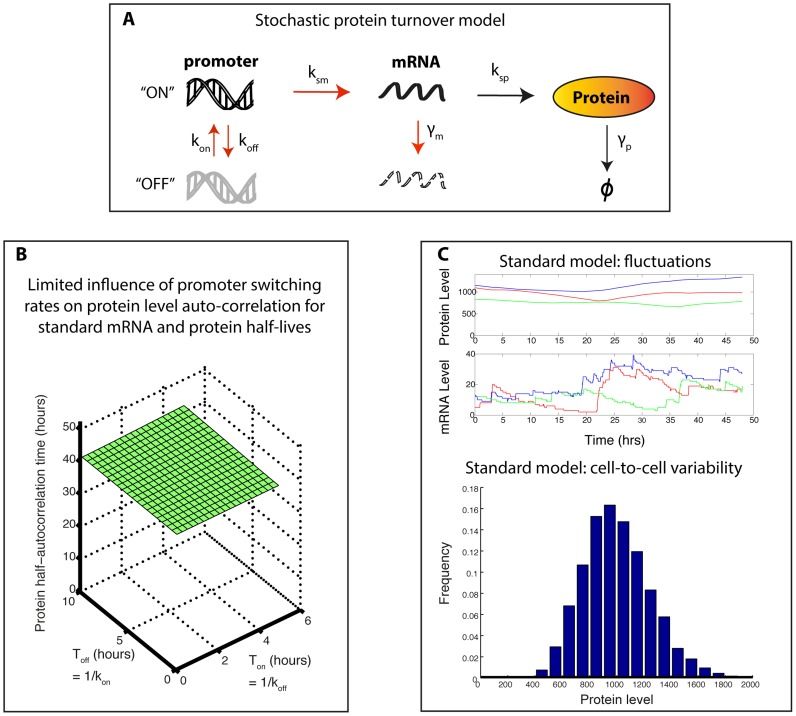
Standard stochastic protein turnover models. (A) Schematic description of the reactions constituting the stochastic protein turnover model. Gene activity switches, mRNA production and degradation (red arrows) are stochastic reactions. Protein synthesis and degradation reactions (black arrows) are deterministic. (B) For typical mRNA and protein half-lives, promoter switching rates have a limited influence on the protein level half-autocorrelation time: using an analytical derivation of the protein level autocorrelation function (see Supplementary Results, Text S1), the protein half-autocorrelation time is plotted against mean promoter switching times. (C) Behavior of a standard stochastic protein turnover model. Promoter switching rates respect typical ranges observed by Suter et al. [36] and lead to a protein level coefficient of variation (CV) of 0.25. See Fig. S3 for more details. Upper plots show three representative single-cell time courses of protein and mRNA levels. Histogram at the bottom displays the corresponding distribution of protein level obtained when simulating a large number of cells for a long duration, corresponding to a snapshot of the cell-to-cell variability expected in a population.

We propose a modeling approach to account for gene expression noise within kinetic models of signal transduction pathways. Following Singh et al. [34], we model protein turnover with stochastic processes describing mRNA level fluctuations, and deterministic processes for protein translation and degradation (random telegraph model, see also [35]–[41]). These processes are integrated into a kinetic model of protein-protein reactions (Fig. 1). While the rates of such stochastic protein turnover models (Fig. 2A) are rarely directly measurable, their value can be constrained by using experimentally measurable data and analytical results (Fig. S2). Recently, significant progress has been made on both experimental and theoretical sides to enable this inference approach [34]–[41]. Importantly, we found that for typical protein and mRNA half-lives, a large set of promoter rate combinations lead to similar fluctuations at the protein level, as characterized by protein level variability (coefficient of variation) and mixing time (half-autocorrelation time). Interestingly, the obtained mixing time is around 40 hours (Fig. 2B), in the middle of the range of experimentally estimated values for twenty endogenous proteins in human cells [26]. Thus, standard stochastic protein turnover models (Fig. 2C) can provide a good approximation of protein fluctuations for most proteins. Only short-lived proteins necessitate particular attention (Fig. S3). This finding is a cornerstone of our approach.

### Modeling stochastic protein turnover in TRAIL-induced apoptosis

We applied this approach to TRAIL-induced apoptosis, using EARM kinetic model [5], [13] to describe protein-protein reactions taking place between TRAIL exposure and cell death commitment. We equipped all native proteins with a default model of stochastic protein turnover, with the exception of proteins with fast turnover, here Flip and Mcl1 [42]–[44]. Details of model construction are given in Text S1 (see dedicated section, Fig. S4 and Tables S1-3). Importantly, all parameters have been constrained based on experimental data and analytical results, with the exception of four parameters (“ON” and “OFF” promoter switching rates, mRNA and protein half-lives for Flip and Mcl1). Note that because of their similar protein and mRNA half-life, we use the same couple of promoter switching rates for both proteins in our exploration of parameter space. This drastically limited the number of introduced degrees of freedom and made it possible to systematically explore realistic ranges for remaining parameters. To study the influence of stochastic protein turnover on fractional killing and reversible resistance, we sought to confront our model with existing quantitative data about TRAIL-induced apoptosis in HeLa cells. Those experiments, described in detail later, can be classified into two groups based on the type of information they contain: 1) quantification of the variability in cell fate, 2) characterization of the transient memory in cell state. While previous approaches using ODE models with distributions for initial protein levels (capturing a static description of cell-to-cell variability) [13], [25] are potentially able to reproduce the first type of data, a dynamic view on cell-to-cell variability as proposed in our model is needed to account for both types of data. We adopted the following strategy: first, search for models able to reproduce observations on cell fate variability; and second test whether valid models can robustly predict observed behaviors where transient memory matters.

### Stochastic protein turnover models predict transient memory in cell sensitivity to TRAIL and CHX

**Figure 3:**
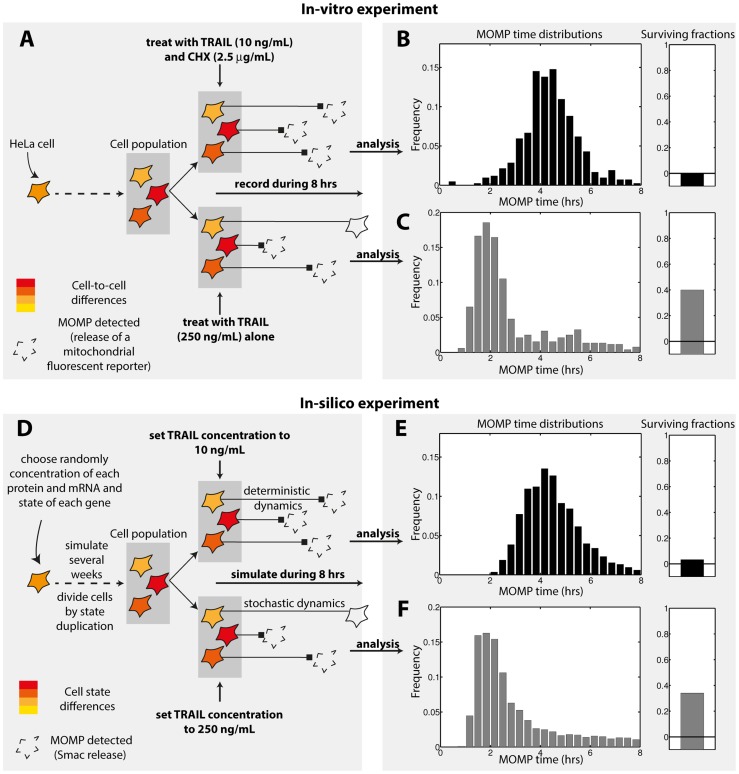
Cell fate variability in TRAIL-induced apoptosis. (A–C) Cell fate variability experiments performed in [13]. (A) HeLa cell populations were treated with either 10 ng/mL of TRAIL and 2.5 µg/mL of cycloheximide (CHX) or 250 ng/mL of TRAIL alone. Cells were tracked during 8 hours by live-cell microscopy and MOMP time was detected via mitochondrial release of a fluorescent reporter. (B–C) Histograms of MOMP times and surviving fractions observed for treatment with (B) TRAIL and CHX or (C) TRAIL alone. (D–F) In-silico reproduction of those experiments with our “fitted” model (i.e. the parameterization in the explored parameter space region giving the best agreement for cell fate variability data, described in Tables S1-3). (D) Simulations (see Supplementary Methods in Text S1 for details). (E–F) Results for the (E) TRAIL and CHX or (F) TRAIL alone treatments. For the latter case, representative model trajectories are given in Fig. S9.

**Figure 4:**
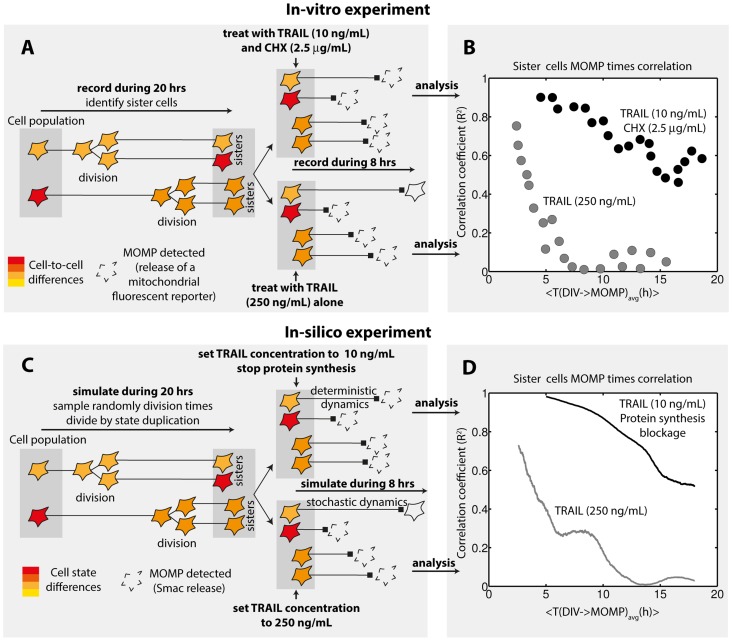
Transient cell fate inheritance in TRAIL-induced apoptosis. (A–B) Experiments measuring correlation of MOMP times between sister cells performed in [13]. (A) HeLa cells were recorded from 20 hours before treatment as in Fig. 3A. Sister cells were identified to permit comparison of their fate. (B) Quantification of cell fate inheritance was realized by computing the correlation between sister cells MOMP time as a function of the duration between division and MOMP (averaged between sisters). (C–D) In-silico reproduction of those experiments with the model of Fig. 3. (C) Description. (D) Quantification of cell fate inheritance was applied to simulation results as in (B). See Supplementary Methods in Text S1 for details.

Using live-cell microscopy, Spencer et al. [13] investigated the fate of hundreds of cells after exposure to TRAIL and CHX (10 ng/mL and 2.5 µg/mL, Fig. 3A). All cells undergo MOMP with a highly variable delay (from 2 to 8 hours, Fig. 3B). To study cell fate inheritance, the authors also recorded 20 hours before treatment to identify sister cells (Fig. 4A). They were found to have highly correlated MOMP times (correlation coefficient close to 1 for recently divided cells, about 0.5 for older sisters - Fig. 4B, black curve). Here, the MOMP time distribution provides a quantification of the cell fate variability, while MOMP time correlations between sister cells also give information on the transient memory in cell state. Within our framework, *in-silico* reproduction of those experiments is straightforward (Figs. 3D and 4C), enabling us to investigate possible origins of transient cell fate inheritance. We first asked if the observed cell fate variability could be reproduced. In the model, it is only determined by protein levels at treatment time (behavior is deterministic as synthesis is assumed to be fully blocked by CHX and noise in signaling reactions is neglected), and differences between sister cells are only caused by protein synthesis noise occurring between division and treatment (in agreement with the fact that recently divided sisters died almost synchronously, we assumed an equal repartition of protein content at division). We found that excellent agreement with observed MOMP time variability can be obtained (Fig. 3E). Further analysis revealed that such agreement requires Flip and Mcl1 protein half-life to be short and to fall within a narrow range (between 0.3 and 0.6 hours, Fig. S5-C). This model prediction is consistent with previous measurements in HeLa cells (30 and 40 minutes for Flip short isoform and Mcl1 respectively, [42], [43]). In contrast, Flip and Mcl1 mRNA half-life and promoter switching rates are not strongly constrained, probably because their influence on cell fate is limited by the rapid protein level decrease caused by synthesis blockade. We then asked whether our extended model also capture transient cell fate inheritance (Fig. 4B). It is the case: fitted models accurately predict the MOMP time correlation between sister cells (Fig. 4D - black curve, Fig. S5-C). Of note, assuming standard promoter switching rates for Flip and Mcl1 (but accounting for their short mRNA and protein half-life - this parameterization will later be referred as the “non-fitted” model) already provides a good agreement for both MOMP time distribution and MOMP time correlation between sister cells (Fig. S6). This non-trivial result shows that the speed at which the sensitivity to TRAIL and CHX fluctuates in single cells is well captured and thus suggests that our generic approach permits to describe fluctuations of protein levels with sufficient accuracy.

### Large, rare fluctuations of short-lived anti-apoptotic proteins explain fractional killing and predict transient cell fate inheritance

**Figure 5:**
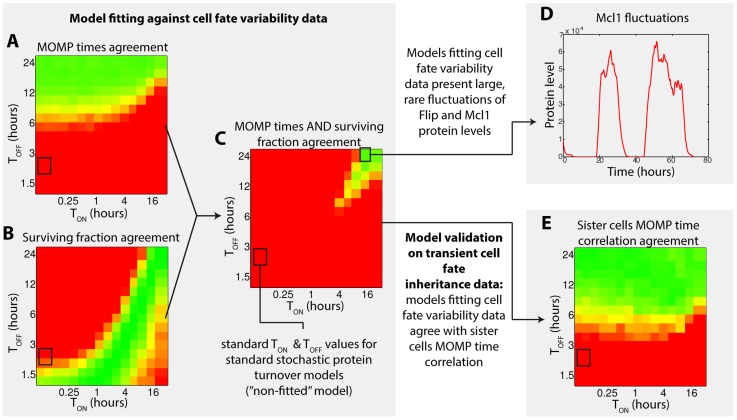
Model fitting to cell fate variability data predicts large, rare fluctuations of Flip/Mcl1 and transient cell fate inheritance. (A–C) Agreement between model prediction and experimental data for (A) death (i.e. MOMP) time distribution, (B) surviving fraction after 8 hours, and (C) both together, for treatment by TRAIL alone (250 ng/mL), as a function of Flip/Mcl1 promoter switching times (other parameters as in Table S1). (D) Representative protein level fluctuations of Mcl1 described by a stochastic protein turnover model allowing good agreement for both MOMP time distribution and surviving fraction. This model has been used for Figs. 3,4,6 and 7. (E) Model-data agreement for MOMP time correlation between sister cells. For (A), (B), (C) and (E), agreement quality increases from red to green. The quantification algorithm is detailed in Supplementary Methods (Text S1).

Spencer and colleagues repeated this experiment but treated cells with TRAIL alone (250 ng/mL). In this condition, an important fraction of cells died fast (MOMP in ∼2 hours) but 40% were still alive after 8 hours (Fig. 3C), illustrating the fractional killing property. Also, cell fate inheritance between sister cells was markedly changed: only young sister cells that underwent MOMP rapidly were importantly correlated (Fig. 4B, grey curve). We asked whether the observed cell fate variability, including fractional killing, could be reproduced *in-silico*. Within our modeling assumptions, absence of co-treatment with CHX makes a fundamental difference: as synthesis continues, the effect of gene expression noise during TRAIL-induced apoptosis could be investigated, and comparison with the TRAIL and CHX condition is insightful. Strikingly, we found that quantitative agreement for both MOMP time distribution and surviving fraction could be obtained (Fig. 3F). Robustness analysis showed that rates of the Flip and Mcl1 stochastic protein turnover model, and particularly promoter switching rates, are in this case strongly constrained. Interestingly, MOMP time distribution and surviving fraction constrain those values differently (Fig. 5A–B), resulting in an narrow ranges for their values: agreement for both observations together is obtained only when promoter switching rates are both low (Fig. 5C). Such low switching rates lead to large, rare fluctuations of protein levels (Fig. 5D). Those atypical fluctuations phenotypes are expected to leave a signature at the population level: the shape of the protein level distribution would be bimodal rather than resembling a lognormal distribution (Fig. S7). This property is thus a model prediction. Those fluctuations are likely to impact how the fate of sister cells diverge with time. Thus, we asked whether the model could also account for the observed fast loss of cell fate inheritance. Remarkably, the fitted models accurately and robustly predict MOMP time correlations between sister cells (Figs. 4D and 5E). As mentioned earlier, the same couple of promoter switching rates was used for Flip and Mcl1 during exploration, but further analysis showed that assuming low promoter switching rates for Mcl1 alone was sufficient to obtain quantitative agreement for MOMP distributions surviving fractions, and that sister cells MOMP time correlations were still correctly predicted (Fig. S8). Thus, comparison with transient cell fate inheritance data supports that large, rare fluctuations of Mcl1 could be responsible for the observed cell fate variability.

### Accounting for stochastic protein turnover predicts reversible resistance

**Figure 6:**
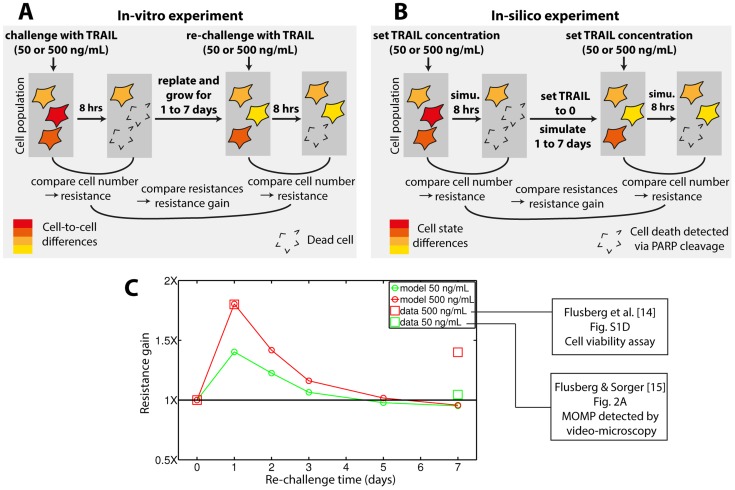
Reversible resistance in repeated TRAIL treatments. (A) Schematic description of the ‘repeated TRAIL’ experiments performed in [14], [15] to characterize reversible resistance in HeLa cells. (B) In-silico reproduction of these experiments with our model (details in Supplementary Methods, Text S1). (C) Resistance gains in surviving cells relative to naïve cells as a function of time between the two TRAIL treatments. Data are shown for experimental observations [14], [15] and model predictions (our study). Comparison of resistance gain experimental measurements between the two TRAIL doses (500 and 50 ng/mL for [14] and [15] respectively) should be done with care, as the measurement and quantification method differed.

Recently, reversible resistance was observed among various cell lines [14]. Cell populations were submitted to two consecutive TRAIL treatments. The duration between treatments was varied from 1 day to 1 week (Fig. 6A). One-day survivors were significantly more resistant than the initial population, but such resistance was significantly decreased or even lost in one-week survivors. Thus, cells surviving a first TRAIL treatment are transiently resistant. Remarkably, *in-silico* reproduction of those (Fig. 6B) showed that our model predicts the presence of reversible resistance (Fig. 6C): one-day survivors exhibit a dose-dependent increase of resistance to a second TRAIL treatment, which disappears after 3 to 5 days. This is surprising since our model does not include induced regulation mediated by survival pathways. Moreover, the presence of reversible resistance is a robust property of the model as it is also obtained when assuming standard promoter switching rates for Mcl1 and Flip (“non-fitted” model, Fig. S6). However, agreement with related experimental data [14], [15] is only qualitative. While we cannot exclude that model parameterizations allowing a quantitative agreement exist, it might be needed to include additional mechanisms such as survival pathways induction to explain the observed sustained resistance gain after one week when treating cells with a high TRAIL dose [14].

### Molecular determinants of fractional killing and reversible resistance

**Figure 7:**
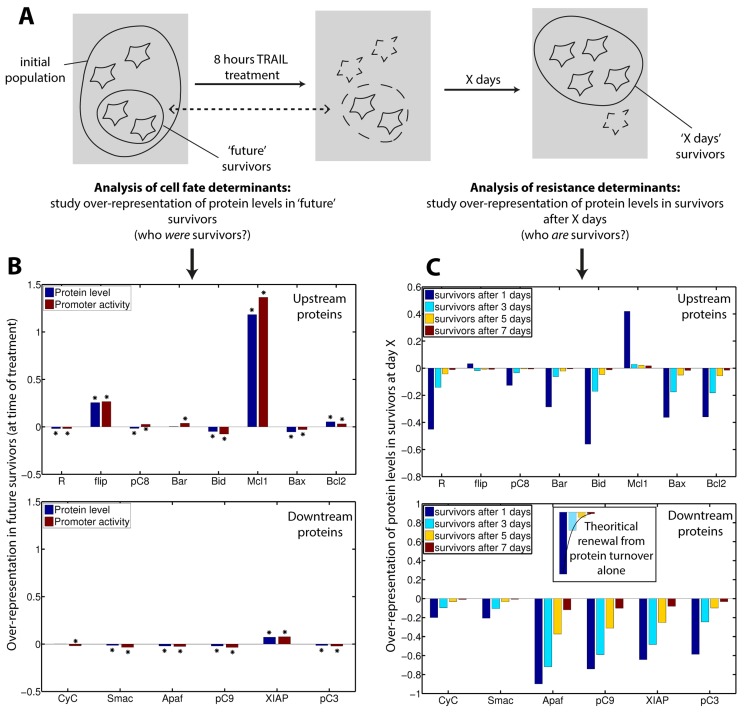
Molecular determinants of cell fate and resistance to repeated TRAIL treatments. (A) Cartoon illustrating that the determinants of cell fate and resistance can be studied by analyzing the over-representation of protein levels in ‘future survivors’ (cells that will still be alive after treatment) at the time of treatment, and in surviving cells at day X, respectively. (B) Cell fate determinants analysis: over-representation (compared to initial population) of protein level (blue) and promoter activity (red) at the time of treatment in ‘future survivors’. Asterisks mark differences that passed a 5% significance test. (C) Resistance determinants analysis: over-representation of protein levels in surviving cells at day X. Inset illustrates the recovery kinetics expected from protein turnover only (i.e. in absence of significant selection effect or residual signaling activity). Therefore, deviation from such kinetics indicates the presence of a selection effect or residual signaling activity.

What are the mechanisms behind cell escape to TRAIL-induced apoptosis, either on the short-term (fractional killing) or the long-term (reversible resistance)? Using the fact that *in-silico*, all protein, mRNA levels and gene activity states can be monitored in single cells, we investigated those questions at the molecular level. To study the influence of pre-existing differences on cell fate, we compared at the time of stimulation the sub-population of ‘future survivors’ with the whole population (Fig. 7A–B). Future survivors strongly stood out by their Mcl1 protein level and gene activity state (Fig. 7B). Flip also appeared to play an important role in determining cell decision, and smaller but significant effect was also seen for Bid, Bax, Bcl2 and XIAP. Although it is a good predictor of cell fate, initial Mcl1 gene activity status does not completely determine survival: neither all Mcl1 “ON” cells survived nor all Mcl1 “OFF” cells died. Thus, pre-existing differences in protein levels and promoter activities are major determinant of cell fate but stochastic events in gene expression occurring during signal transduction also play a role. While timing of death for cells treated with TRAIL and CHX appeared to be multi-factorial [13], our results suggest that cell survival is predominantly determined by Mcl1 (Fig. 7B). This important role of Mcl1 is robustly predicted. Indeed, it also holds for the “non-fitted” model, which assume standard promoter switching rates for all proteins, including Mcl1 and Flip (Fig. S6).

To investigate the determinants of reversible resistance, we tracked the temporal evolution of protein levels in surviving cells (Fig. 7 A&C). The protein level composition of one day survivors contrasts with the protein content observed in future survivors: almost all protein levels differ importantly from the naïve population composition, while that was the case only for Mcl1 and Flip in future survivors. This is expected as all proteins are partly activated during signal transduction, leading to a higher degradation (active forms have a shorter half-life). Therefore, the distinction between the causes of cell survival and the consequences of partial apoptosis induction cannot be easily resolved by the sole observation of protein levels in survivors. When signaling stops, recovery of protein levels is expected to follow exponential kinetics governed by the turnover rate (Fig. 7C, inset – see the death receptor (R), pro-caspase 8, Bar and Bid). Deviation from such kinetics indicates either the persistence of signaling reactions that continue to consume proteins (it is the case for Apaf, pro-caspase 9 and XIAP, as further analysis confirmed) or is a consequence of important selection. Indeed, while Mcl1 and Flip should recover normal levels in a few hours in absence of selection because of their high turnover rate, Mcl1 levels (but not Flip levels) are still strongly higher than in naive cells one day after TRAIL treatment, consistently with previous observations on the relative selection strength that operated on them. Together, those results indicate that recovery phenotypes in surviving cells result from a complex interplay of three distinct effects: selection during apoptosis, transcriptional noise and protein turnover as a driving force tending to reset protein levels to their initial, pre-stimulus distribution, and long-term residual signaling activity. This explains why it is difficult to understand the recovery process and justifies the use of modeling to disentangle the various contributions.

## Discussion

Rehm et al. and Spencer et al. [13], [20] made two insightful observations about TRAIL-induced apoptosis. First, recently born sister cells died almost synchronously when treated with TRAIL and a protein synthesis inhibitor, while in contrast, unrelated cells died after highly variable durations. This demonstrated that TRAIL signaling is mostly deterministic when protein synthesis is blocked and that the timing of death is determined by the cell internal state at the time of treatment. Second, they observed that such synchrony in sister cells death is gradually lost as the time between division and treatment increases. This showed that the cell 'TRAIL sensitivity state' (the part of cell internal state involved in death timing determination) naturally fluctuates over a dozen of hours. In addition, the modeling results in ref. [13] highly suggested that such state is mainly composed by the various levels of the proteins acting in the extrinsic apoptosis pathway. In parallel, important progress on the characterization of the stochasticity in gene expression has been made: the two-state transcriptional bursting model was shown to permit high accuracy and several approaches to infer its parameters were proposed, enabling the quantitative modeling of protein fluctuations in single cells [34]–[40].

### Modeling protein fluctuations in TRAIL-induced apoptosis

In this study, we merged those two approaches by integrating such stochastic models of gene expression within an existing kinetic model of TRAIL-induced apoptosis [5] in a systematic and principled manner. Doing so provides advantages compared to previous approaches to account for cell-to-cell variability in protein levels [13], [25]. First, variability is not considered as an “input” parameter but arises naturally from stochastic fluctuations. The dynamics of this variability is thus intrinsically represented within the system, allowing investigating the effects of transient memory in protein levels. Second, the influence of protein synthesis noise during TRAIL-induced apoptosis could also be investigated. Importantly, we followed a parsimonious parameterization strategy, motivated by the fact that fluctuations of long-lived proteins are rather insensitive to the precise kinetics of transcriptional bursting, enabling us to equip most proteins (long-lived proteins) with reasonably accurate fluctuation models even in absence of gene expression data for each and every promoter. The sister cells experiment for which cells were treated with TRAIL and CHX provided ideal data to validate our modeling approach: in that case, behavior is mostly deterministic as soon as treatment starts and only fluctuations occurring before treatment are responsible for death time variability and de-correlation between sister cells. Moreover, gene regulation via survival pathways induction is ineffective as protein synthesis is blocked. Because our model was able to quantitatively reproduce the MOMP time distribution and then accurately predicted sister cells correlation, our modeling approach appears as a promising tool to investigate the effect of protein fluctuations on signal transduction, despite the limitations inherent to its simplicity (for example, stochastic gene expression events were assumed to be independent between proteins, neglecting the fact that levels of different proteins can be partially correlated [25], [26], possibly because of common transcription factors or coordinated chromatin-state transitions). Of note, good agreement was readily obtained when assuming for Flip and Mcl1 standard promoter switching rates, but short protein and mRNA half-lives - in agreement with available knowledge (“non-fitted” model, Fig. S6). Finally, the transposition of fluctuation timescales from individual proteins into ‘TRAIL sensitivity states’ is not trivial: while in our model, stable proteins levels are mixed in about 40 hours, cells were switching between 'fast dying' and 'slow dying' phenotypes more rapidly (about 10–15 hours). As combinatorial effects are at play, mechanistic models of protein-proteins reactions are needed to link protein-level timescales with more high-level phenotypic transitions [45].

### Questioning the role of survival pathways

Several studies reported that TRAIL can induce survival pathways [17]–[19]. How such induced changes affect signal transduction and eventually stop apoptotic signaling remains unclear. On the other hand, the contribution of constitutive protein synthesis noise, which is responsible for pre-existing differences between cells, has not been evaluated. Although it does not exclude the existence of other mechanisms, an important result of our study is that fractional killing can be obtained without assuming any TRAIL-induced regulation. Alternatively, we find that because of its fast turnover, constitutive expression of the Mcl1 protein has the potential to rescue cells from TRAIL apoptotic signaling. In this context, solely accounting for protein fluctuations within the TRAIL apoptosis pathway predicts the fractional killing property (Figs. 3 and S6). While our results challenge current opinion on the role of survival pathways in TRAIL-induced apoptosis, they are consistent with observations made on wild type HeLa cells that neither blocking NF-κB response nor inhibiting the Akt pathway do significantly change the surviving cell fraction after TRAIL treatment [46], [47]. The pivotal role for Mcl1 in TRAIL-induced apoptosis predicted by our model is consistent with the recent finding that Mcl1 silencing by shRNA in HeLa cells completely sensitize cells to TRAIL [48]. While moderate fluctuations of Mcl1 levels were sufficient to obtain fractional killing, a quantitative agreement with the Spencer et al. [13] single-cell data (MOMP time distribution and surviving fraction) required large and rare Mcl1 fluctuations, caused by rare switches between long periods of gene activity or inactivity. Interestingly, in that case, the observed rapid loss of MOMP time correlation between sister cells quantitatively emerged from model simulations. Flip is often mentioned as a key factor in cell resistance to TRAIL [49], but in our model Flip has less impact on cell survival than Mcl1. Consistently, Lemke et al. [48] silencing experiments demonstrated a dominant role for Mcl1 and a synergy with Flip. However, our model might under-estimate the role of Flip: the representation of DISC-related events in EARM is simple and thus does not account for recent biological findings, including the stoichiometry between its components [50], [51]. Improving how DISC assembly is modeled might thus be needed to elucidate the precise role of Flip in fractional killing and reversible resistance, especially for cell lines that express higher Flip amounts than HeLa.

### Origins of reversible resistance: joint effect of selection and stochastic protein turnover

A second significant result reported here is that our model predicts the phenomenon of reversible resistance, showing that constitutively noisy protein synthesis, protein-protein interactions and protein degradation are by themselves sufficient to explain a dose-dependent, significant increase of resistance in recent survivors and its gradual loss within 3–5 days. This result is consistent with the observation that Nf-κB blockade does not change resistance acquisition after TRAIL treatment [14] (in MCF10A cells; HeLa cells have not been tested). *In-silico* analysis at the molecular level revealed that reversible resistance as predicted by the model was shaped by a complex interplay between 1) selection based on protein levels and transcriptional activity, 2) protein turnover and 3) residual signaling activity. As opposed to the death process, which involves a sharp and complete activation of effector caspases, our results suggest that recovery in cells that did not commit to death is a slow and complex process. While one should not conclude from our results that parallel activation of survival pathways by TRAIL plays no role in reversible resistance, our results show that the sole contribution of protein level fluctuations occurring within the extrinsic apoptosis pathway can partly lead to reversible resistance. Thus, protein fluctuations should be accounted for to gain quantitative insights into reversible resistance.

### Accounting for gene expression noise appears necessary to investigate signal transduction

While here we focused on TRAIL-induced apoptosis, our modeling approach is generic and can be applied to other signal transduction pathways. Our results showed that even in absence of induced gene regulation, gene expression noise interacts with signaling dynamics on a non-trivial manner. Thus, even in contexts where the influence of induced gene-regulation is indisputable, its sound quantification probably requires to investigate first the role of constitutive gene expression noise. Only then models could be enriched parsimoniously with well-characterized regulatory links until all observations are successfully explained. Significant advances to allow such detailed characterization of gene regulation occurred recently [31], [52], [53]. Following such approaches could significantly extend the reach of models of signal transduction towards accurate, single-cell level description of populations submitted to varying signaling contexts over multiple cell generations.

## Methods

### Modeling stochastic protein turnover

Denoting 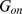 and 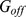 the two states of the promoter, 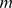 the number of mRNAs and 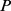 the protein level, the stochastic protein turnover model comprises the following reactions: 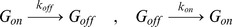 (Gene activity switches).


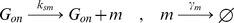
 (Transcription and mRNA degradation).


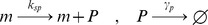
 (Translation and protein degradation).

We interpret the four first reactions as stochastic reactions and the translation and protein degradation reactions as deterministic reactions. Thus, the model state is given by a Boolean 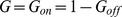, an integer 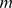 and a continuous variable 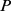. The statistical properties of this system can be investigated analytically by writing the corresponding chemical master equation, which describes the temporal evolution of the state joint probability distribution. While the obtained equations cannot be solved analytically, it can be used to derive moments of the underlying distribution by applying generating functions techniques, as was done by Paszek [39]. We next give the analytical expressions of several moments of interest:
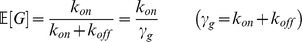


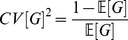


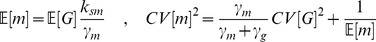


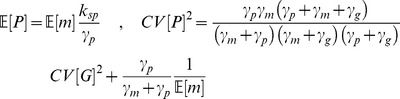



While those expressions characterize the steady-state distribution of the model, and thus can be compared to snapshot measurements of a cell population, they do not provide information about how fast single cells move with time within those distributions. Such information can be obtained by computing the autocorrelation function. Its expression and derivation are given in Supplementary Results (Text S1).

### Modeling TRAIL-induced apoptosis and protein fluctuations

We used the EARM model described in Spencer et al. [13] to represent protein-protein reactions. No kinetic rates were changed, except the rate of pC6 cleavage by C3, set to zero to remove the feedback loop. Degradation rates of non-native forms were modified according to biological knowledge (Table S3). The effect of those changes is discussed in SI Appendix. Rates of the standard stochastic protein turnover models and rates of Flip&Mcl1 models were determined following an algorithm constructed to incorporate constraints based on biological knowledge (Fig. S4). Their values are given in Table S1-2. Notably, for comparability and consistency with the findings of Spencer et al. [13], we kept their mean protein values, even if for a few proteins new data were available, as for Mcl1 for example [54], provided that the order of magnitude is the same.

### In-silico “sister cells” and “repeated TRAIL” experiments

The simulation procedure is detailed in SI Appendix. Briefly, mother cell states (promoter activity, mRNA and protein levels) were chosen by Monte-Carlo sampling and the two sister cells were constructed by duplication of the mother cell state. Promoter activity and mRNA fluctuations were simulated using an implementation of the Gillespie algorithm. For the ODEs governing evolution of all protein levels, the Semi-Implicit Extrapolation method was used. MOMP was considered to have occurred when half of mitochondrial Smac has been released. For all simulations, at least 10^4^ pairs of sister cells were simulated. Quantitative comparison of simulation results with related experimental data (Fig. 5) was achieved by computing a measure of model-data agreement described in Supplementary Methods (Text S1). For repeated TRAIL simulations, a naïve population of 10^4^ cells was obtained as in the sister cells experiment. Cells divided after a gaussian cell cycle duration (mean 27 hours, standard deviation 3 hours). Death was detected via cPARP levels as in Gaudet et al. [25].

## Supporting Information

Figure S1 **Simplified view of the TRAIL-induced apoptosis pathway.** Only the type of each protein-protein interaction (activation or inhibition) is represented. A diagram displaying the detail of protein complexes and activated forms can be find in [5].
(PDF)

Figure S2 **Numerical simulation and analytical characterization of stochastic protein turnover models.** A stochastic protein turnover model is defined by six rates: the promoter activity switching rates, the mRNA production and degradation rates, the protein per mRNA synthesis rate and the protein degradation rate. Numerical simulations can be used to simulate temporal fluctuations in single cells. When a population of cells is simulated, the cell-to-cell variability can be studied. After some time, cell-to-cell variability reaches a steady state. Analytical calculations on the stochastic protein turnover model provide expressions characterizing the steady-state variability (moments of the steady-state distributions), but also fluctuations (autocorrelation functions). Complete expressions are given in Text S1.
(PDF)

Figure S3 **Fluctuations of protein levels caused by transcriptional bursting are smoothed out for long-lived proteins.** Comparison of protein level coefficient of variation (**A**) and half-autocorrelation time (**C**) as a function of transcriptional bursting rates for two situations: a short-lived protein and mRNA (half-lives of 2 and 1 hours, resp.) and a long-lived protein and mRNA (27 and 9 hours, resp.). Other rates of the stochastic protein turnover model are chosen such that mean protein and mRNA level are the same (1000 and 17). Combinations of Ton and Toff values ranging from 0.1 to 5 hours and 0.1 to 10 hours respectively were tested. Ton and Toff are mean ON and OFF time of the gene. (**B and D**) Representation of the range of values obtained for all models tested in (A) and (C).
(PDF)

Figure S4 **Building stochastic protein turnover models for TRAIL-induced apoptosis.** Routine followed to choose rates of all 17 native proteins in the EARM kinetic model of TRAIL-induced apoptosis. Typical values from multi-genes studies in mammalian cells are used to constrain rate values. Specific attention is given to Flip and Mcl1 because they are known to be short-lived, and thus more prone to exhibit large variations.
(PDF)

Figure S5 **Stochastic protein turnover models captures fluctuations of cell sensitivity to TRAIL and CHX.** (**A**) Best found agreement between model and data for MOMP times distribution in the +CHX condition. Obtained for Flip and Mcl1 model rates such that protein/mRNA half-life and mean ON/OFF promoter activity duration equaled 0.4/1 and 1.9/3.1 hours respectively. See Supplementary Methods (Text S1) for quantification of model data agreement. (**B**) Best found agreement between model and data for MOMP time correlation between sisters in the +CHX condition. Obtained for Flip and Mcl1 model rates such that protein/mRNA half-life and mean ON/OFF promoter activity duration equaled 0.3/1 and 0.35/24 hours respectively. (**C**) Influence of Flip and Mcl1 model rates on Model-Data agreement in the +CHX condition. For each parameter, we plot the model to data distance corresponding to the best model when all other three parameters are varied.
(PDF)

Figure S6 **The “non-fitted” model quantitatively predicts TRAIL+CHX single-cell data and lead to fractional killing and reversible resistance for TRAIL alone treatments.** In the non-fitted model, Flip and Mcl1 promoter switching rates are standard (Ton  = 0.1 hours and Toff  = 2.6 hours) but the short-half life of their mRNA and protein is accounted for (2 hours and 0.5 hours respectively). We reproduce here for this model all the results presented in the main text for the “fitted” model. Quantitative agreement is obtained for TRAIL + CHX single cell data from Spencer et al. [13] (MOMP time distribution and sister cell MOMP time correlations). No quantitative agreement is obtained in the case of TRAIL alone treatments, but the existence of fractional killing and reversible resistance is nevertheless predicted. Note that because fewer cells were simulated compared to main text figures (50000 instead of 10^5^ for sister cell experiments), sister correlation curves appears slightly noisier.
(PDF)

Figure S7 **Mcl1 and Flip fluctuations for standard or “fitted” promoter switching rates.** For the top frame, promoter switching rates are standard (as in Fig. 2). Because mRNA and protein half-lives are short, protein level fluctuates more rapidly and the steady-state distribution is changed (it is wider and the mode is in 0) compared to the standard stochastic protein turnover model (Fig. 2). On the bottom frame, the steady-state distribution becomes bimodal because the promoter switching rates are low compared to mRNA and protein degradation. In both cases, fluctuations and distribution are shown for Mcl1; they are similar for Flip as only the protein synthesis rate changes to account for a different mean protein level.
(PDF)

Figure S8 **Large, rare fluctuations of Mcl1 alone are sufficient to explain cell fate variability and transient inheritance in both conditions.** While Flip and Mcl1 protein and mRNA half-lives were the same as for the “fitted” model (0.4 and 1.0 hours respectively), only the Mcl1 promoter was assumed to have low switching rates (Ton and Toff are 16 and 24 hours resp.). The switching rates of the Flip promoter were assumed to be standard (Ton  = 0.1 hours and Toff  = 2.6 hours). All the results presented in the main text for the “fitted” model are reproduced here. Note that because fewer cells were simulated compared to main text figures (50000 instead of 10^5^ for sister cell experiments), sister correlation curves appears slightly noisier.
(PDF)

Figure S9 **Representative single-cell trajectories before and after TRAIL treatment for the “fitted” model.** Trajectories for two dying and two surviving cells (after 12 hours of TRAIL treatment) are shown. T-marked arrows denote the time of TRAIL addition (250 ng/mL), D-marked arrows denote the time of death commitment (MOMP). mRNA (lower left of each panel) and native form protein levels (upper left of each panel) are shown for pro-caspase 8, Bid and Mcl1. Levels of activated caspase 8, truncated Bid and activated caspase 3 are also shown (upper right of each panel), as well as the ratio of released Smac and of cleaved PARP (lower right of each panel).
(PDF)

Figure S10 **Robustness of short-term model behavior regarding the presence/absence of feedback loop and the degradation of active forms.** Model-Data agreement is shown for MOMP time distributions, surviving fractions and sisters correlation of MOMP time in both treatment conditions for model variants when the C3->C6->C8 feedback loop is either present/absent and the default active forms half-life is 27, 15, 5 or 2 hours. Significant model-data deviation is seen only for the fastest active forms degradation.
(PDF)

Figure S11 **Resistance gain in one day survivors is robust regarding the presence/absence of feedback loop and the degradation of active forms.** In-silico repeated TRAIL experiment (as in Fig. 6) was repeated for variants of the “fitted” model regarding presence/absence of the C3->C6->C8 feedback loop and the default active forms half-life. Resistance gain in one-day survivors is shown. Simulations were repeated 4 times with 10^4^ cells, error bars indicate standard deviation of estimated resistance gain between replicates.
(PDF)

Figure S12 **Long-term population survival is not possible with the feedback loop and stable active forms.** Evolution of alive cell number in populations treated in-silico as in Fig. 6, for model several variants regarding presence/absence of the C3->C6->C8 feedback loop and the default active forms half-life.
(PDF)

Table S1 **Standard stochastic protein turnover models.**
(DOCX)

Table S2 **Specific stochastic protein turnover models.**
(DOCX)

Table S3 **Non-native form degradation.**
(DOCX)

Text S1 **Supplementary results and supplementary methods.**
(DOCX)
