# Supplementary figures and images for: Modeling Dynamics of Cell-to-Cell Variability in TRAIL-Induced Apoptosis Explains Fractional Killing and Predicts Reversible Resistance
Source: PLoS Comput Biol. 2014 Oct 23;10(10):e1003893. doi: 10.1371/journal.pcbi.1003893 (PMC4207462; doi:10.1371/journal.pcbi.1003893)

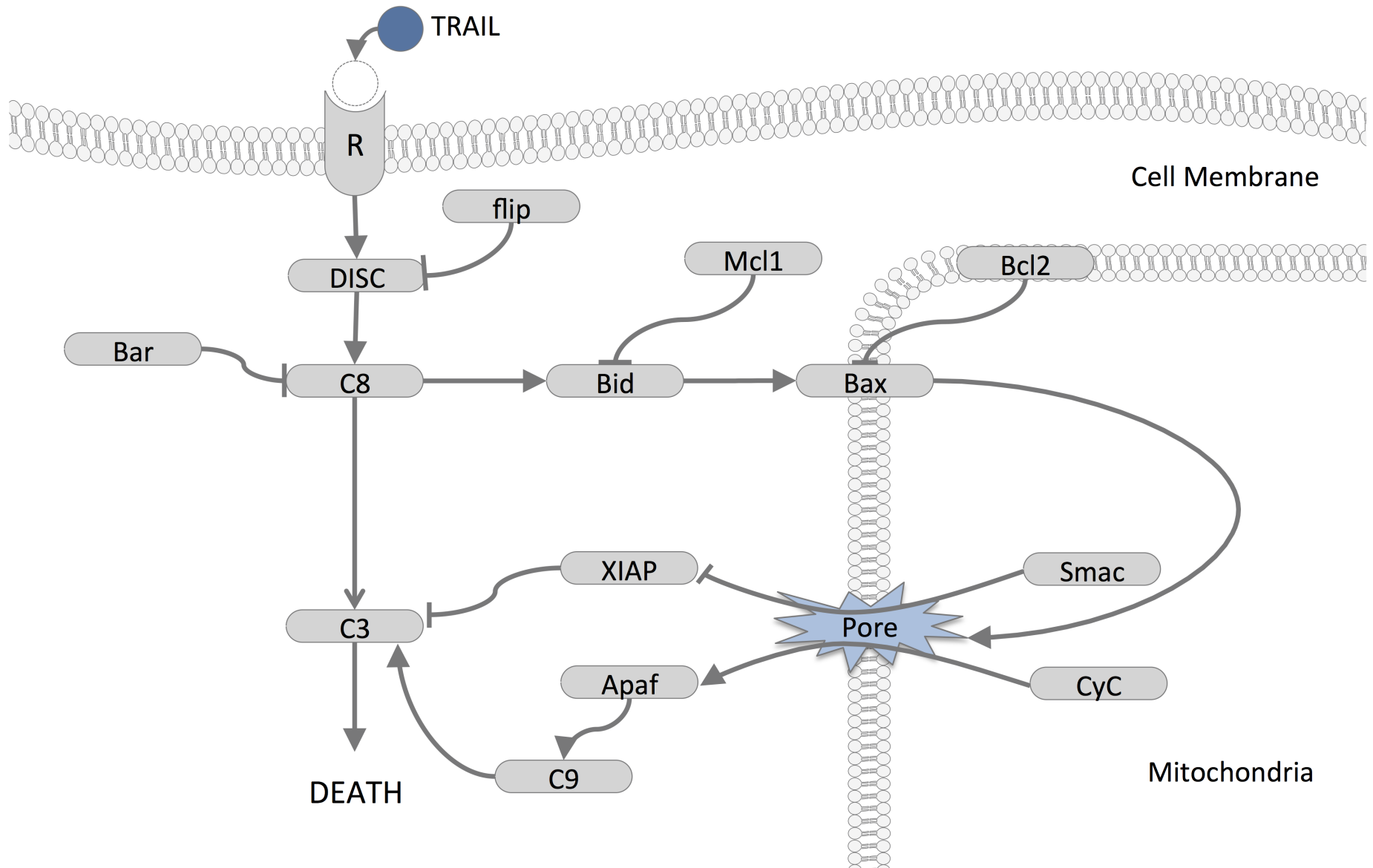

Supplement: Figure S1 — Simplified view of the TRAIL-induced apoptosis pathway. Only the type of each protein-protein interaction (activation or inhibition) is represented. A diagram displaying the detail of protein complexes and activated forms can be find in [5]. (PDF) [file pcbi.1003893.s001.pdf]

## Early dying cell

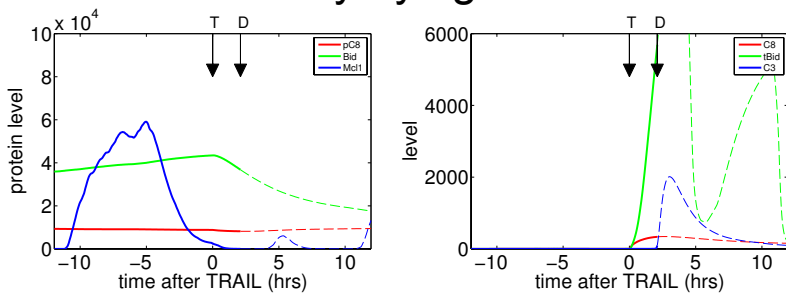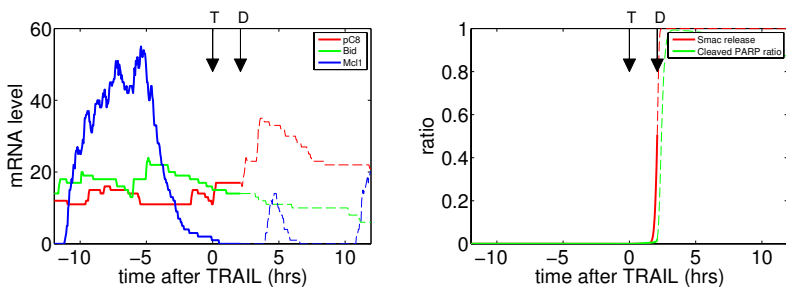

## Late dying cell

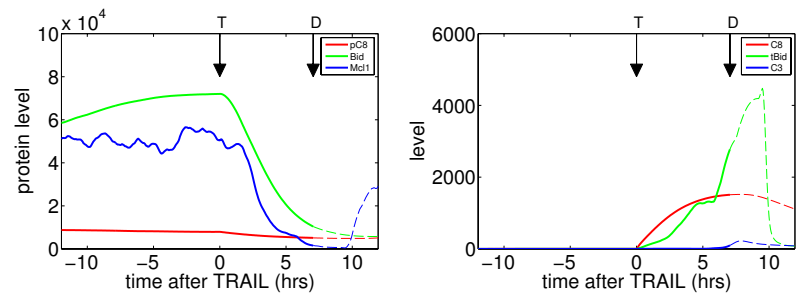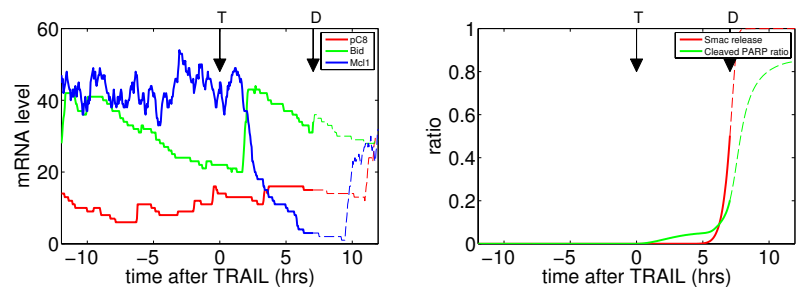

## Surviving cell

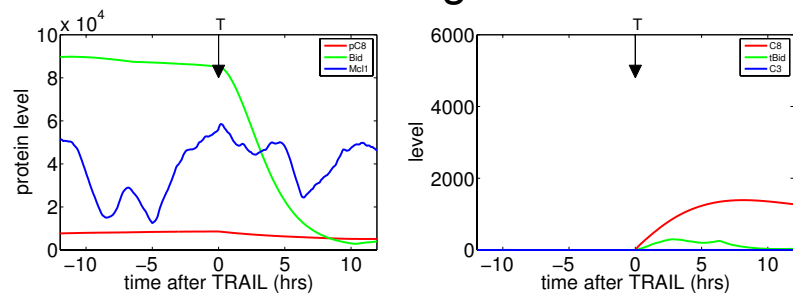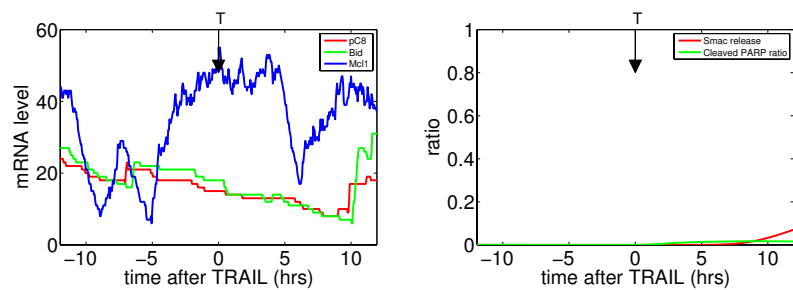

## Surviving cell

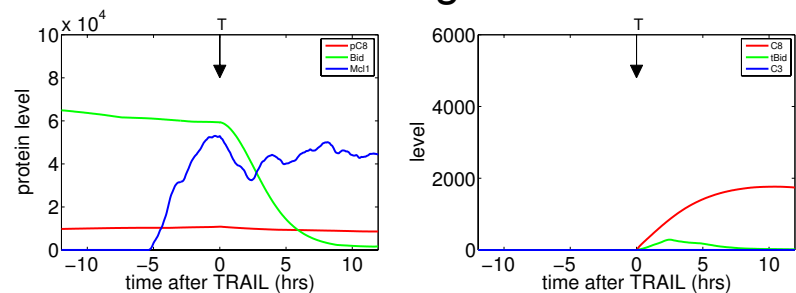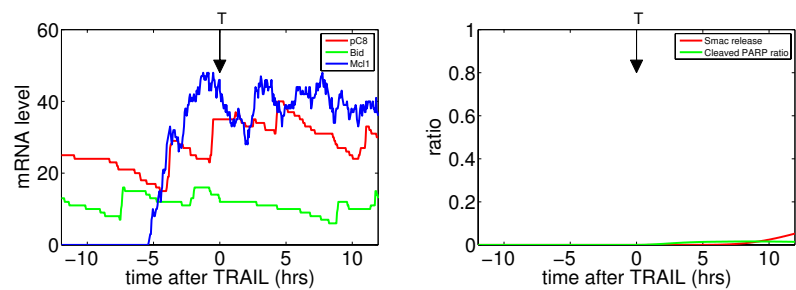

Supplement: Figure S9 — Representative single-cell trajectories before and after TRAIL treatment for the “fitted” model. Trajectories for two dying and two surviving cells (after 12 hours of TRAIL treatment) are shown. T-marked arrows denote the time of TRAIL addition (250 ng/mL), D-marked arrows denote the time of death commitment (MOMP). mRNA (lower left of each panel) and native form protein levels (upper left of each panel) are shown for pro-caspase 8, Bid and Mcl1. Levels of activated caspase 8, truncated Bid and activated caspase 3 are also shown (upper right of each panel), as well as the ratio of released Smac and of cleaved PARP (lower right of each panel). (PDF) [file pcbi.1003893.s009.pdf]

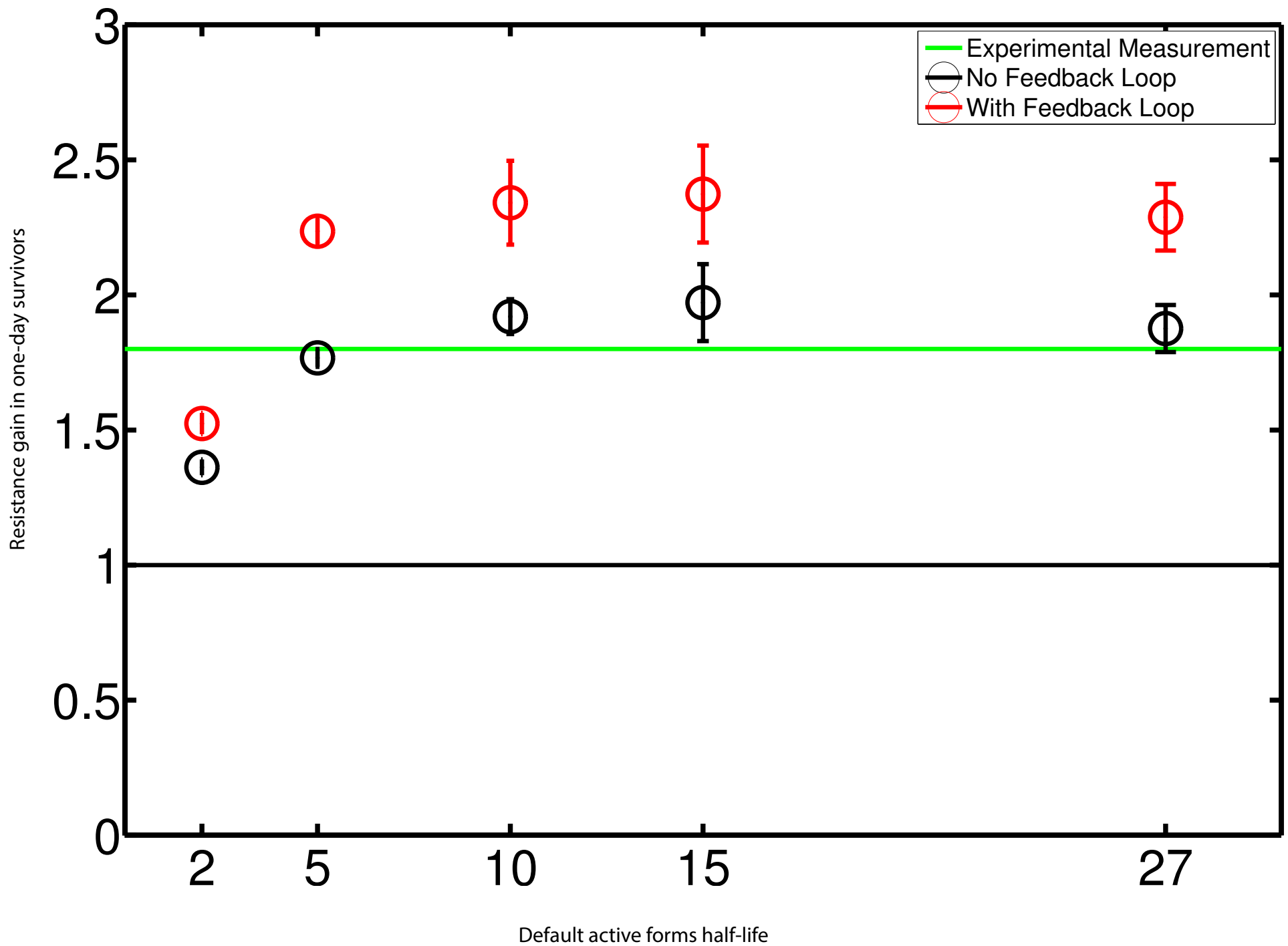

Supplement: Figure S11 — Resistance gain in one day survivors is robust regarding the presence/absence of feedback loop and the degradation of active forms. In-silico repeated TRAIL experiment (as in Fig. 6) was repeated for variants of the “fitted” model regarding presence/absence of the C3->C6->C8 feedback loop and the default active forms half-life. Resistance gain in one-day survivors is shown. Simulations were repeated 4 times with 104 cells, error bars indicate standard deviation of estimated resistance gain between replicates. (PDF) [file pcbi.1003893.s011.pdf]

With feedback loop

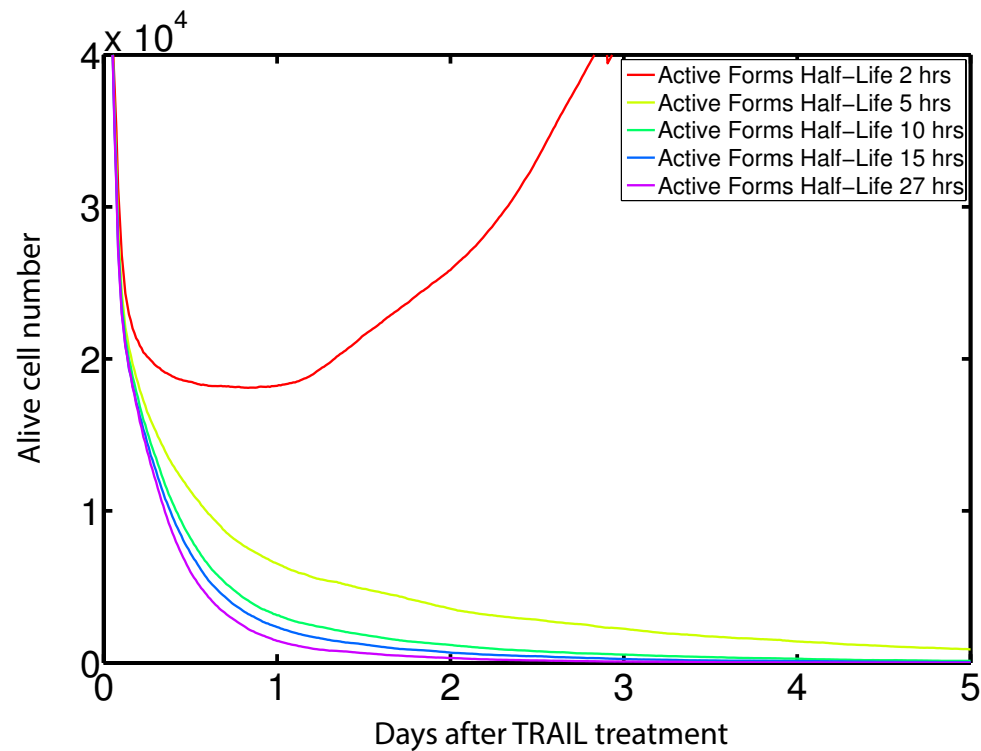

Without feedback loop

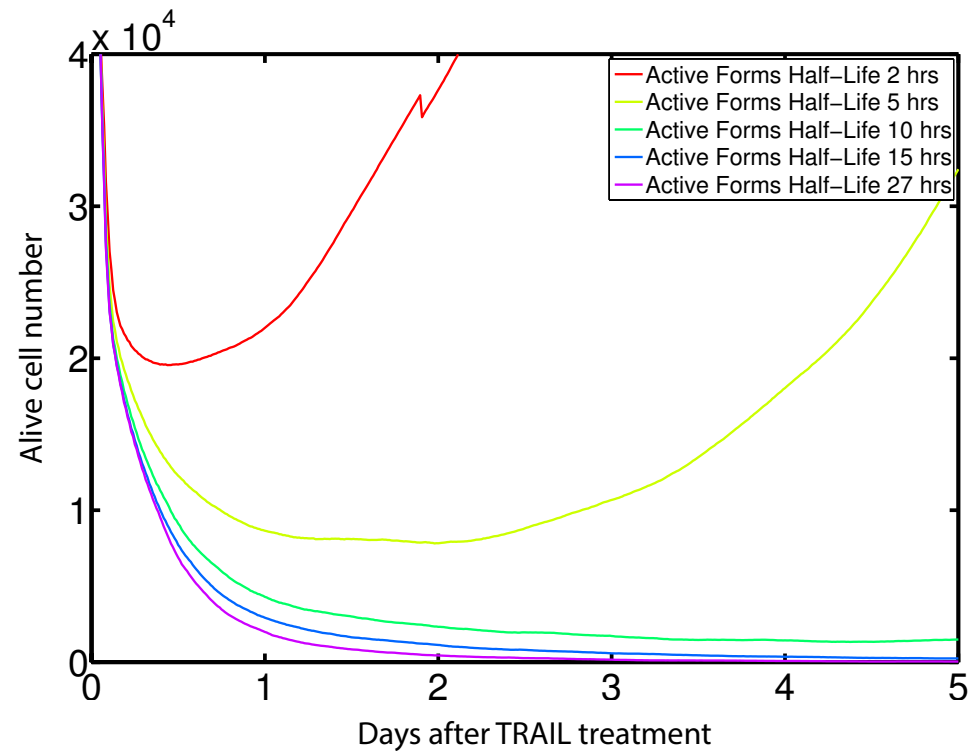

Supplement: Figure S12 — Long-term population survival is not possible with the feedback loop and stable active forms. Evolution of alive cell number in populations treated in-silico as in Fig. 6, for model several variants regarding presence/absence of the C3->C6->C8 feedback loop and the default active forms half-life. (PDF) [file pcbi.1003893.s012.pdf]
