# Supplementary material for: Modeling Dynamics of Cell-to-Cell Variability in TRAIL-Induced Apoptosis Explains Fractional Killing and Predicts Reversible Resistance
Source: PLoS Comput Biol. 2014 Oct 23;10(10):e1003893. doi: 10.1371/journal.pcbi.1003893 (PMC4207462; doi:10.1371/journal.pcbi.1003893)
Supplement: Figure S2 — Numerical simulation and analytical characterization of stochastic protein turnover models. A stochastic protein turnover model is defined by six rates: the promoter activity switching rates, the mRNA production and degradation rates, the protein per mRNA synthesis rate and the protein degradation rate. Numerical simulations can be used to simulate temporal fluctuations in single cells. When a population of cells is simulated, the cell-to-cell variability can be studied. After some time, cell-to-cell variability reaches a steady state. Analytical calculations on the stochastic protein turnover model provide expressions characterizing the steady-state variability (moments of the steady-state distributions), but also fluctuations (autocorrelation functions). Complete expressions are given in Text S1. (PDF) [file pcbi.1003893.s002.pdf]

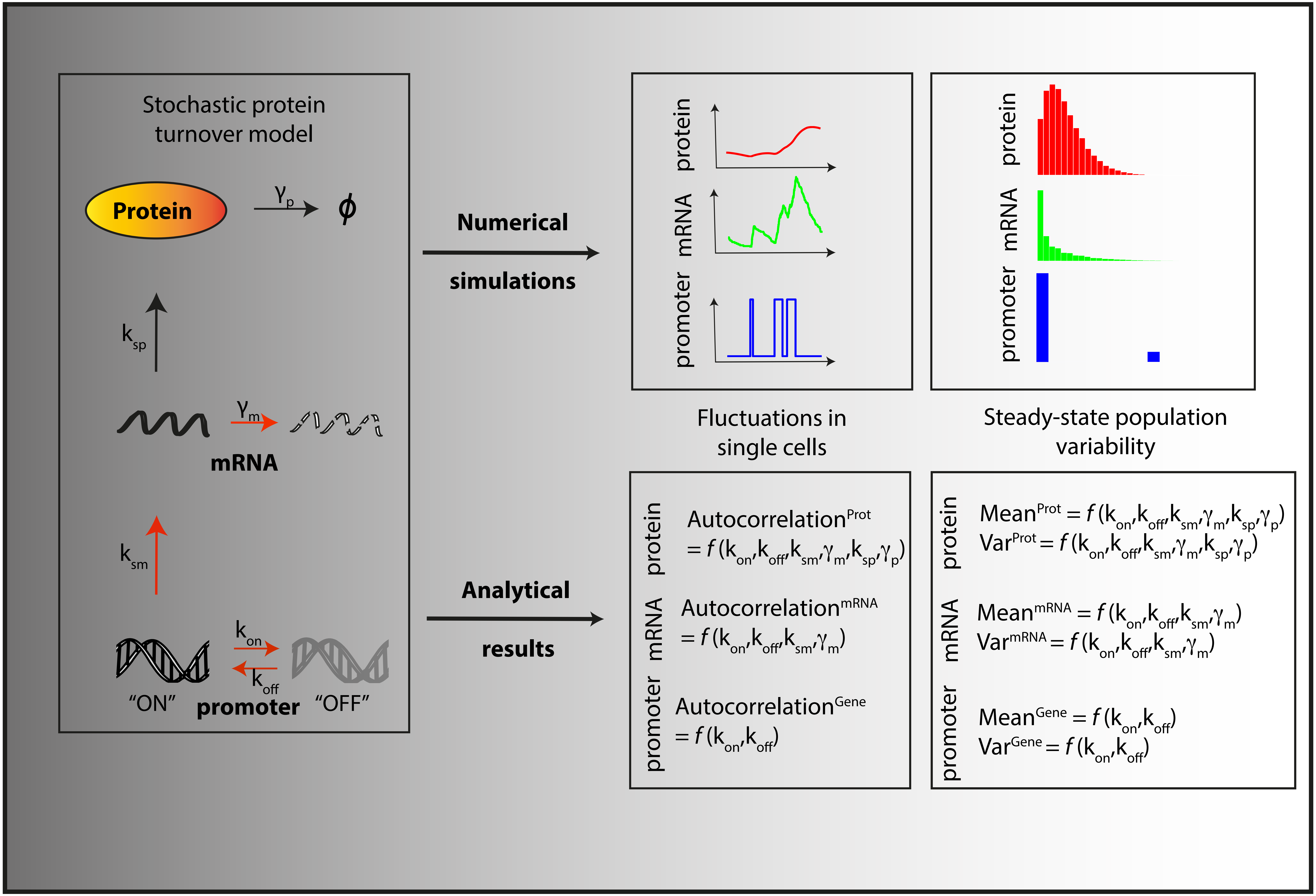

Stochastic protein turnover model
Protein
γ_p
ϕ
k_{sp}
γ_m
mRNA
k_{sm}
k_{on}
k_{off}
"ON"
promoter
"OFF"
Numerical simulations
Analytical results
protein
mRNA
promoter
Fluctuations in single cells
Steady-state population variability
$\text{Autocorrelation}^{Prot} = f(k_{on}, k_{off}, k_{sm}, \gamma_m, k_{sp}, \gamma_p)$
$\text{Autocorrelation}^{mRNA} = f(k_{on}, k_{off}, k_{sm}, \gamma_m)$
$\text{Autocorrelation}^{Gene} = f(k_{on}, k_{off})$
$\text{Mean}^{Prot} = f(k_{on}, k_{off}, k_{sm}, \gamma_m, k_{sp}, \gamma_p)$
$\text{Var}^{Prot} = f(k_{on}, k_{off}, k_{sm}, \gamma_m, k_{sp}, \gamma_p)$
$\text{Mean}^{mRNA} = f(k_{on}, k_{off}, k_{sm}, \gamma_m)$
$\text{Var}^{mRNA} = f(k_{on}, k_{off}, k_{sm}, \gamma_m)$
$\text{Mean}^{Gene} = f(k_{on}, k_{off})$
$\text{Var}^{Gene} = f(k_{on}, k_{off})$
