# Supplementary material for: Modeling Dynamics of Cell-to-Cell Variability in TRAIL-Induced Apoptosis Explains Fractional Killing and Predicts Reversible Resistance
Source: PLoS Comput Biol. 2014 Oct 23;10(10):e1003893. doi: 10.1371/journal.pcbi.1003893 (PMC4207462; doi:10.1371/journal.pcbi.1003893)
Supplement: Figure S3 — Fluctuations of protein levels caused by transcriptional bursting are smoothed out for long-lived proteins. Comparison of protein level coefficient of variation (A) and half-autocorrelation time (C) as a function of transcriptional bursting rates for two situations: a short-lived protein and mRNA (half-lives of 2 and 1 hours, resp.) and a long-lived protein and mRNA (27 and 9 hours, resp.). Other rates of the stochastic protein turnover model are chosen such that mean protein and mRNA level are the same (1000 and 17). Combinations of Ton and Toff values ranging from 0.1 to 5 hours and 0.1 to 10 hours respectively were tested. Ton and Toff are mean ON and OFF time of the gene. (B and D) Representation of the range of values obtained for all models tested in (A) and (C). (PDF) [file pcbi.1003893.s003.pdf]

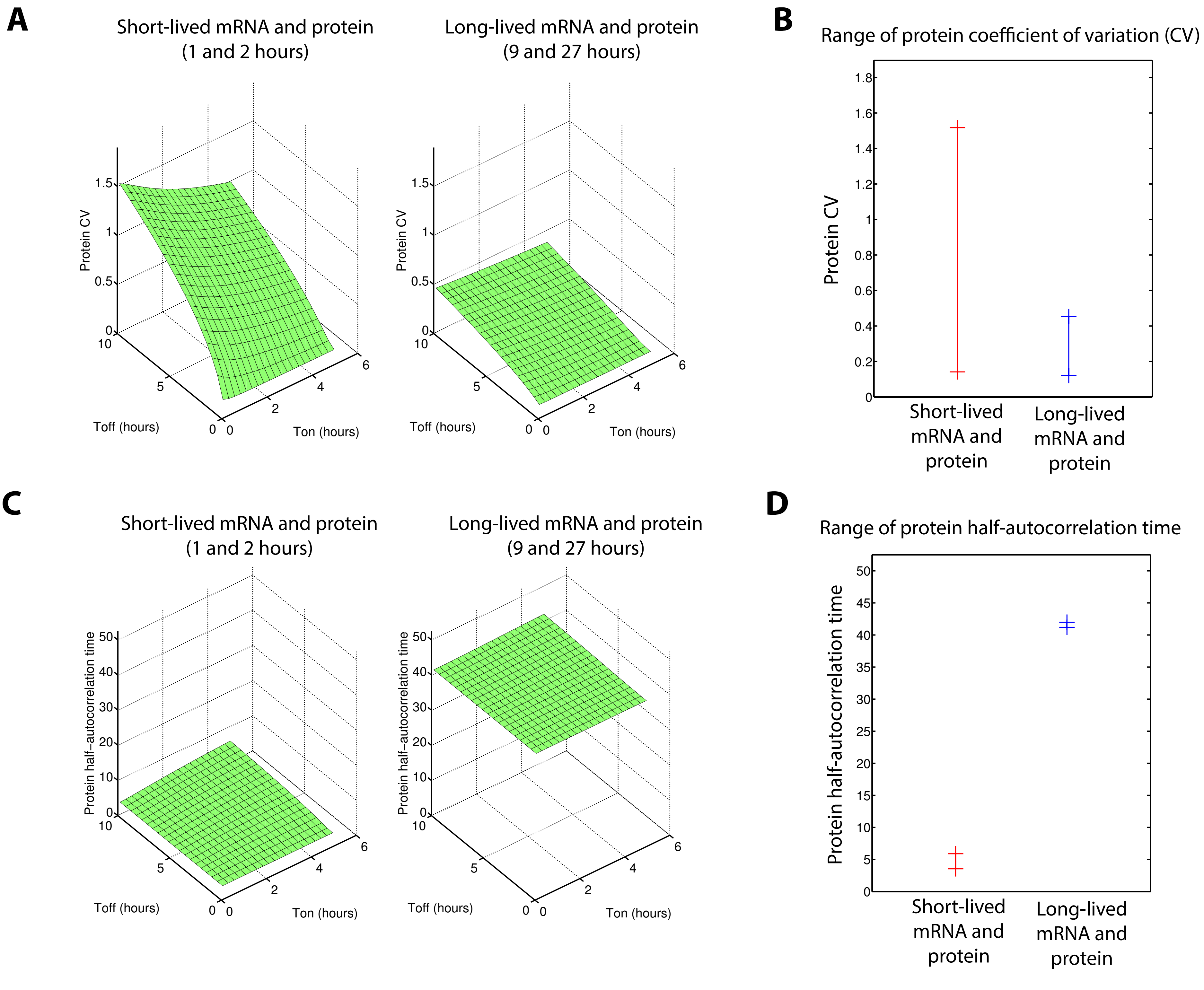
A
Short-lived mRNA and protein
(1 and 2 hours)
Long-lived mRNA and protein
(9 and 27 hours)
Protein CV
Toff (hours)
Ton (hours)
B
Range of protein coefficient of variation (CV)
Protein CV
Short-lived mRNA and protein
Long-lived mRNA and protein
C
Short-lived mRNA and protein
(1 and 2 hours)
Long-lived mRNA and protein
(9 and 27 hours)
Protein half-autocorrelation time
Toff (hours)
Ton (hours)
D
Range of protein half-autocorrelation time
Protein half-autocorrelation time
Short-lived mRNA and protein
Long-lived mRNA and protein
