# Supplementary material for: Modeling Dynamics of Cell-to-Cell Variability in TRAIL-Induced Apoptosis Explains Fractional Killing and Predicts Reversible Resistance
Source: PLoS Comput Biol. 2014 Oct 23;10(10):e1003893. doi: 10.1371/journal.pcbi.1003893 (PMC4207462; doi:10.1371/journal.pcbi.1003893)
Supplement: Figure S4 — Building stochastic protein turnover models for TRAIL-induced apoptosis. Routine followed to choose rates of all 17 native proteins in the EARM kinetic model of TRAIL-induced apoptosis. Typical values from multi-genes studies in mammalian cells are used to constrain rate values. Specific attention is given to Flip and Mcl1 because they are known to be short-lived, and thus more prone to exhibit large variations. (PDF) [file pcbi.1003893.s004.pdf]

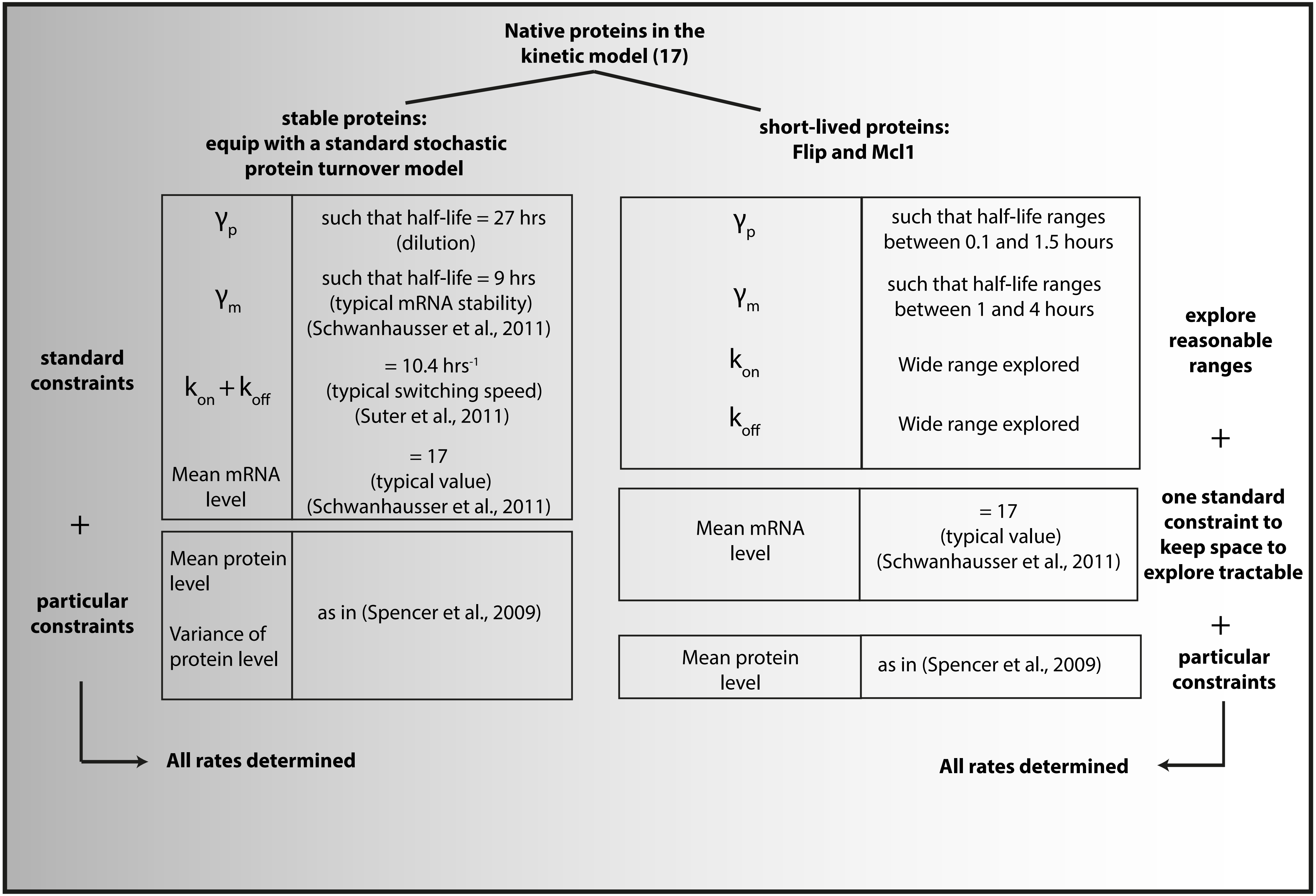
Native proteins in the kinetic model (17)
stable proteins: equip with a standard stochastic protein turnover model
short-lived proteins: Flip and Mcl1
standard constraints
+
particular constraints
γp
such that half-life = 27 hrs (dilution)
γm
such that half-life = 9 hrs (typical mRNA stability) (Schwanhausser et al., 2011)
kon + koff
= 10.4 hrs-1 (typical switching speed) (Suter et al., 2011)
Mean mRNA level
= 17 (typical value) (Schwanhausser et al., 2011)
Mean protein level
Variance of protein level
as in (Spencer et al., 2009)
All rates determined
γp
such that half-life ranges between 0.1 and 1.5 hours
γm
such that half-life ranges between 1 and 4 hours
kon
Wide range explored
koff
Wide range explored
explore reasonable ranges
+
Mean mRNA level
= 17 (typical value) (Schwanhausser et al., 2011)
one standard constraint to keep space to explore tractable
+
Mean protein level
as in (Spencer et al., 2009)
particular constraints
All rates determined
