# Supplementary material for: Modeling Dynamics of Cell-to-Cell Variability in TRAIL-Induced Apoptosis Explains Fractional Killing and Predicts Reversible Resistance
Source: PLoS Comput Biol. 2014 Oct 23;10(10):e1003893. doi: 10.1371/journal.pcbi.1003893 (PMC4207462; doi:10.1371/journal.pcbi.1003893)
Supplement: Figure S5 — Stochastic protein turnover models captures fluctuations of cell sensitivity to TRAIL and CHX. (A) Best found agreement between model and data for MOMP times distribution in the +CHX condition. Obtained for Flip and Mcl1 model rates such that protein/mRNA half-life and mean ON/OFF promoter activity duration equaled 0.4/1 and 1.9/3.1 hours respectively. See Supplementary Methods (Text S1) for quantification of model data agreement. (B) Best found agreement between model and data for MOMP time correlation between sisters in the +CHX condition. Obtained for Flip and Mcl1 model rates such that protein/mRNA half-life and mean ON/OFF promoter activity duration equaled 0.3/1 and 0.35/24 hours respectively. (C) Influence of Flip and Mcl1 model rates on Model-Data agreement in the +CHX condition. For each parameter, we plot the model to data distance corresponding to the best model when all other three parameters are varied. (PDF) [file pcbi.1003893.s005.pdf]

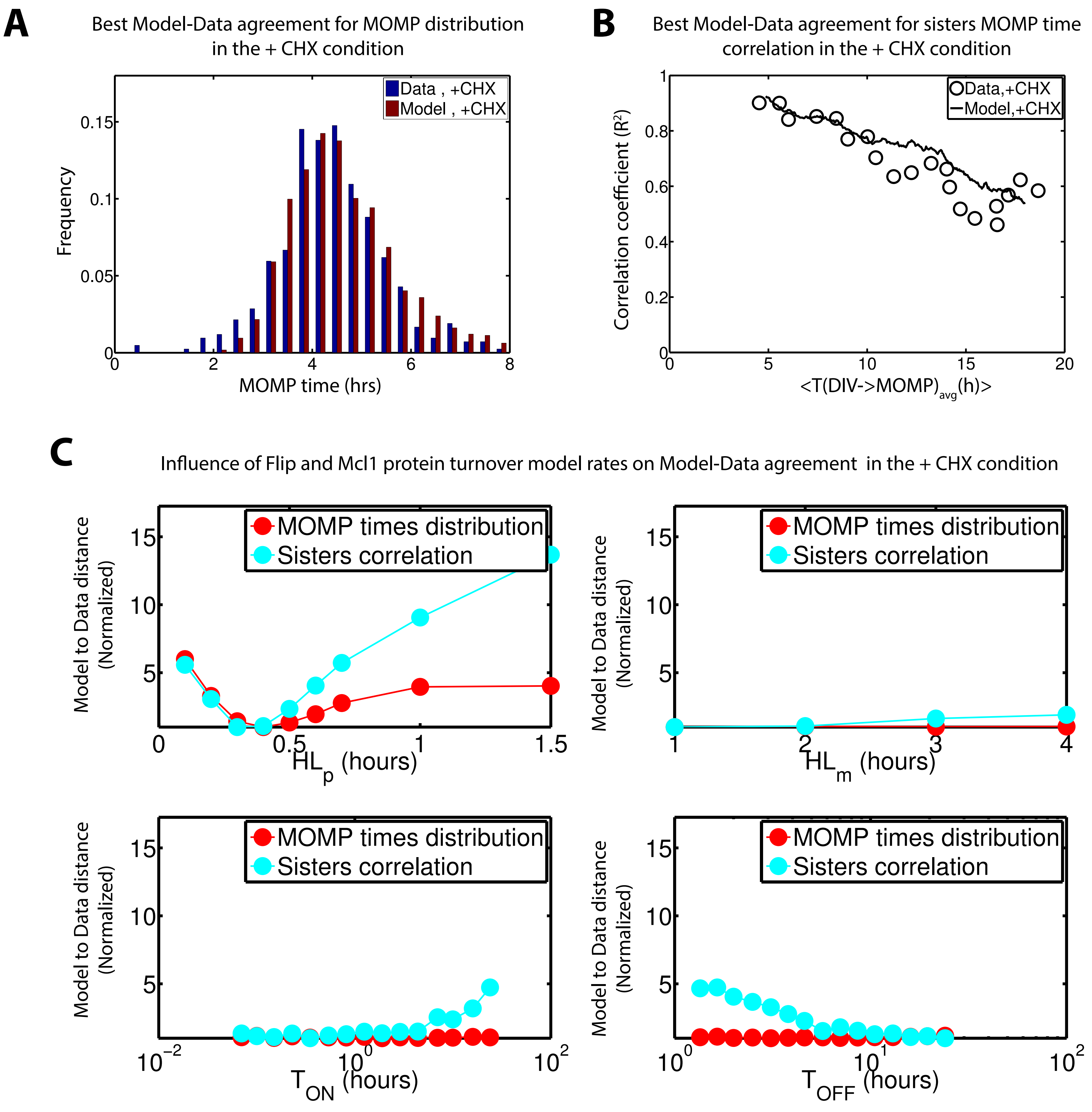
A
Best Model-Data agreement for MOMP distribution in the + CHX condition
Data , +CHX
Model , +CHX
Frequency
0.15
0.1
0.05
0
0
2
4
6
8
MOMP time (hrs)
B
Best Model-Data agreement for sisters MOMP time correlation in the + CHX condition
Data,+CHX
Model,+CHX
Correlation coefficient (R^2)
1
0.8
0.6
0.4
0.2
0
0
5
10
15
20
<T(DIV->MOMP)$_{avg}$(h)>
C
Influence of Flip and Mcl1 protein turnover model rates on Model-Data agreement in the + CHX condition
MOMP times distribution
Sisters correlation
Model to Data distance (Normalized)
15
10
5
0
0.5
1
1.5
HL_p (hours)
MOMP times distribution
Sisters correlation
Model to Data distance (Normalized)
15
10
5
1
2
3
4
HL_m (hours)
MOMP times distribution
Sisters correlation
Model to Data distance (Normalized)
15
10
5
10^{-2}
10^{0}
10^{2}
T_{ON} (hours)
MOMP times distribution
Sisters correlation
Model to Data distance (Normalized)
15
10
5
10^{0}
10^{1}
10^{2}
T_{OFF} (hours)
