# Supplementary material for: Modeling Dynamics of Cell-to-Cell Variability in TRAIL-Induced Apoptosis Explains Fractional Killing and Predicts Reversible Resistance
Source: PLoS Comput Biol. 2014 Oct 23;10(10):e1003893. doi: 10.1371/journal.pcbi.1003893 (PMC4207462; doi:10.1371/journal.pcbi.1003893)
Supplement: Figure S6 — The “non-fitted” model quantitatively predicts TRAIL+CHX single-cell data and lead to fractional killing and reversible resistance for TRAIL alone treatments. In the non-fitted model, Flip and Mcl1 promoter switching rates are standard (Ton = 0.1 hours and Toff = 2.6 hours) but the short-half life of their mRNA and protein is accounted for (2 hours and 0.5 hours respectively). We reproduce here for this model all the results presented in the main text for the “fitted” model. Quantitative agreement is obtained for TRAIL + CHX single cell data from Spencer et al. [13] (MOMP time distribution and sister cell MOMP time correlations). No quantitative agreement is obtained in the case of TRAIL alone treatments, but the existence of fractional killing and reversible resistance is nevertheless predicted. Note that because fewer cells were simulated compared to main text figures (50000 instead of 105 for sister cell experiments), sister correlation curves appears slightly noisier. (PDF) [file pcbi.1003893.s006.pdf]

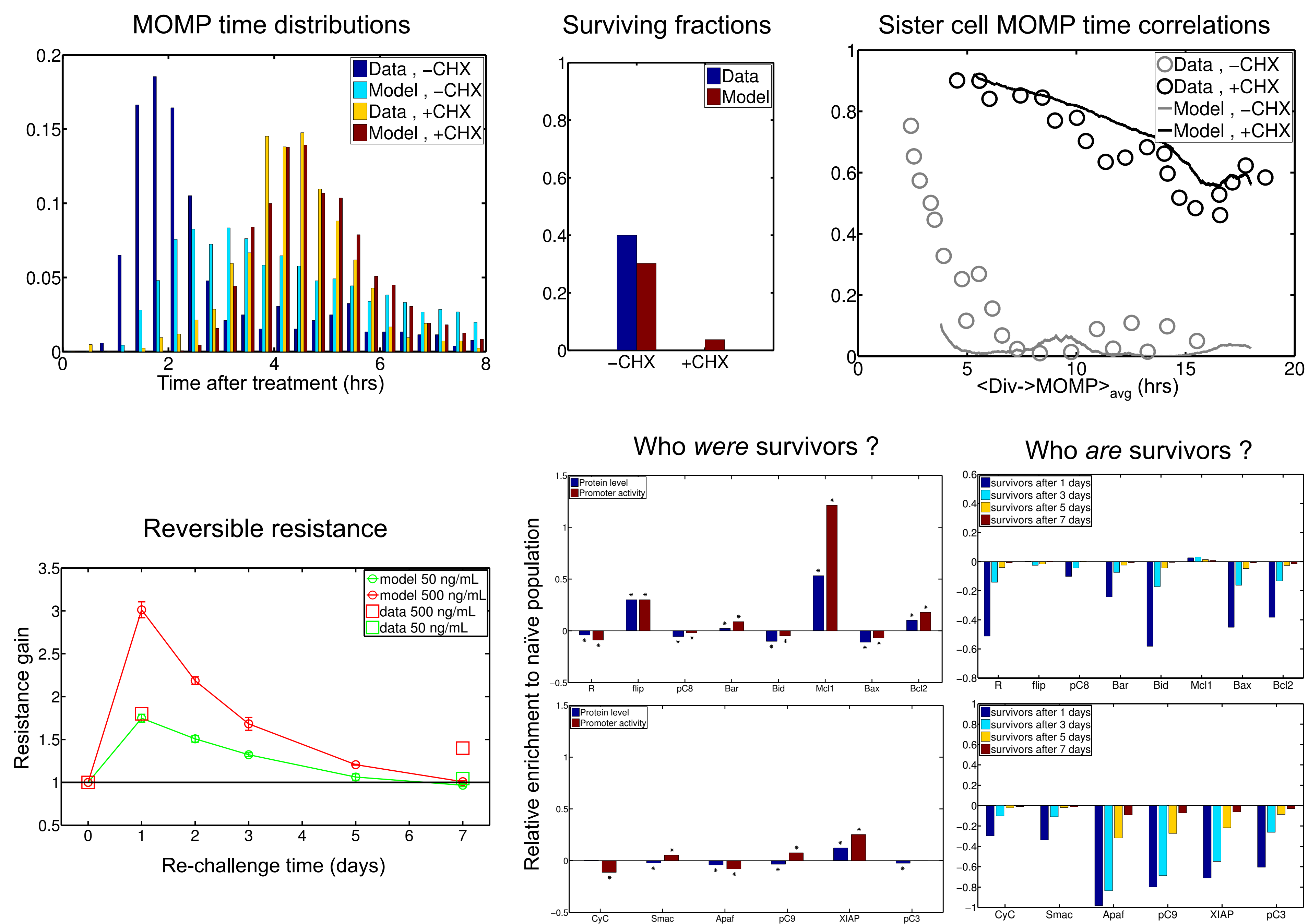

MOMP time distributions
Data , −CHX
Model , −CHX
Data , +CHX
Model , +CHX
Time after treatment (hrs)
Surviving fractions
Data
Model
−CHX
+CHX
Sister cell MOMP time correlations
Data , −CHX
Data , +CHX
Model , −CHX
Model , +CHX
<Div->MOMP>avg (hrs)
Reversible resistance
model 50 ng/mL
model 500 ng/mL
data 500 ng/mL
data 50 ng/mL
Resistance gain
Re-challenge time (days)
Who were survivors ?
Relative enrichment to naive population
Protein level
Promoter activity
R
flip
pC8
Bar
Bid
Mcl1
Bax
Bcl2
CyC
Smac
Apaf
pC9
XIAP
pC3
Who are survivors ?
survivors after 1 days
survivors after 3 days
survivors after 5 days
survivors after 7 days
