# Supplementary material for: Modeling Dynamics of Cell-to-Cell Variability in TRAIL-Induced Apoptosis Explains Fractional Killing and Predicts Reversible Resistance
Source: PLoS Comput Biol. 2014 Oct 23;10(10):e1003893. doi: 10.1371/journal.pcbi.1003893 (PMC4207462; doi:10.1371/journal.pcbi.1003893)

Mcl1 (and Flip) fluctuations for the "non-fitted" model

Standard promoter switching rates

	Mcl1	Flip
protein half-life (hrs)	0.5	
mRNA half-life (hrs)	2	
Ton (hrs)	0.1	
Toff (hrs)	2.6	
protein distribution CV	0.85	
Protein distribution modality	unimodal	

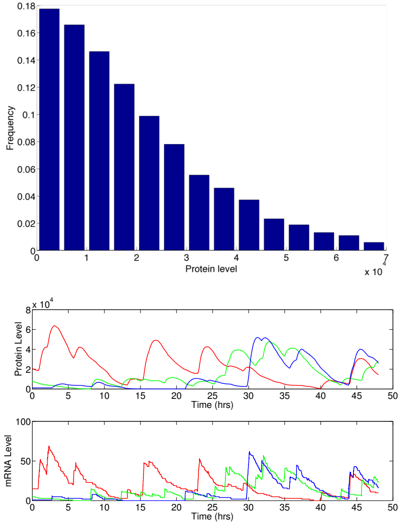

Mcl1 (and Flip) fluctuations for the "fitted" model

Best agreement found with Spencer et al. cell fate variability data

	Mcl1	Flip
protein half-life (hrs)	0.4	
mRNA half-life (hrs)	1	
Ton (hrs)	16	
Toff (hrs)	24	
protein distribution CV	1.15	
Protein distribution modality	bimodal	

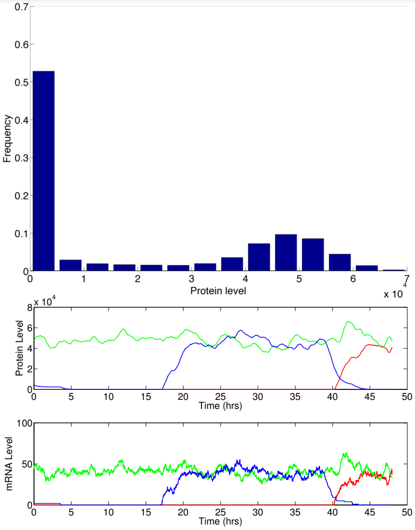

Supplement: Figure S7 — Mcl1 and Flip fluctuations for standard or “fitted” promoter switching rates. For the top frame, promoter switching rates are standard (as in Fig. 2). Because mRNA and protein half-lives are short, protein level fluctuates more rapidly and the steady-state distribution is changed (it is wider and the mode is in 0) compared to the standard stochastic protein turnover model (Fig. 2). On the bottom frame, the steady-state distribution becomes bimodal because the promoter switching rates are low compared to mRNA and protein degradation. In both cases, fluctuations and distribution are shown for Mcl1; they are similar for Flip as only the protein synthesis rate changes to account for a different mean protein level. (PDF) [file pcbi.1003893.s007.pdf]
