# Supplementary material for: Modeling Dynamics of Cell-to-Cell Variability in TRAIL-Induced Apoptosis Explains Fractional Killing and Predicts Reversible Resistance
Source: PLoS Comput Biol. 2014 Oct 23;10(10):e1003893. doi: 10.1371/journal.pcbi.1003893 (PMC4207462; doi:10.1371/journal.pcbi.1003893)
Supplement: Figure S8 — Large, rare fluctuations of Mcl1 alone are sufficient to explain cell fate variability and transient inheritance in both conditions. While Flip and Mcl1 protein and mRNA half-lives were the same as for the “fitted” model (0.4 and 1.0 hours respectively), only the Mcl1 promoter was assumed to have low switching rates (Ton and Toff are 16 and 24 hours resp.). The switching rates of the Flip promoter were assumed to be standard (Ton = 0.1 hours and Toff = 2.6 hours). All the results presented in the main text for the “fitted” model are reproduced here. Note that because fewer cells were simulated compared to main text figures (50000 instead of 105 for sister cell experiments), sister correlation curves appears slightly noisier. (PDF) [file pcbi.1003893.s008.pdf]

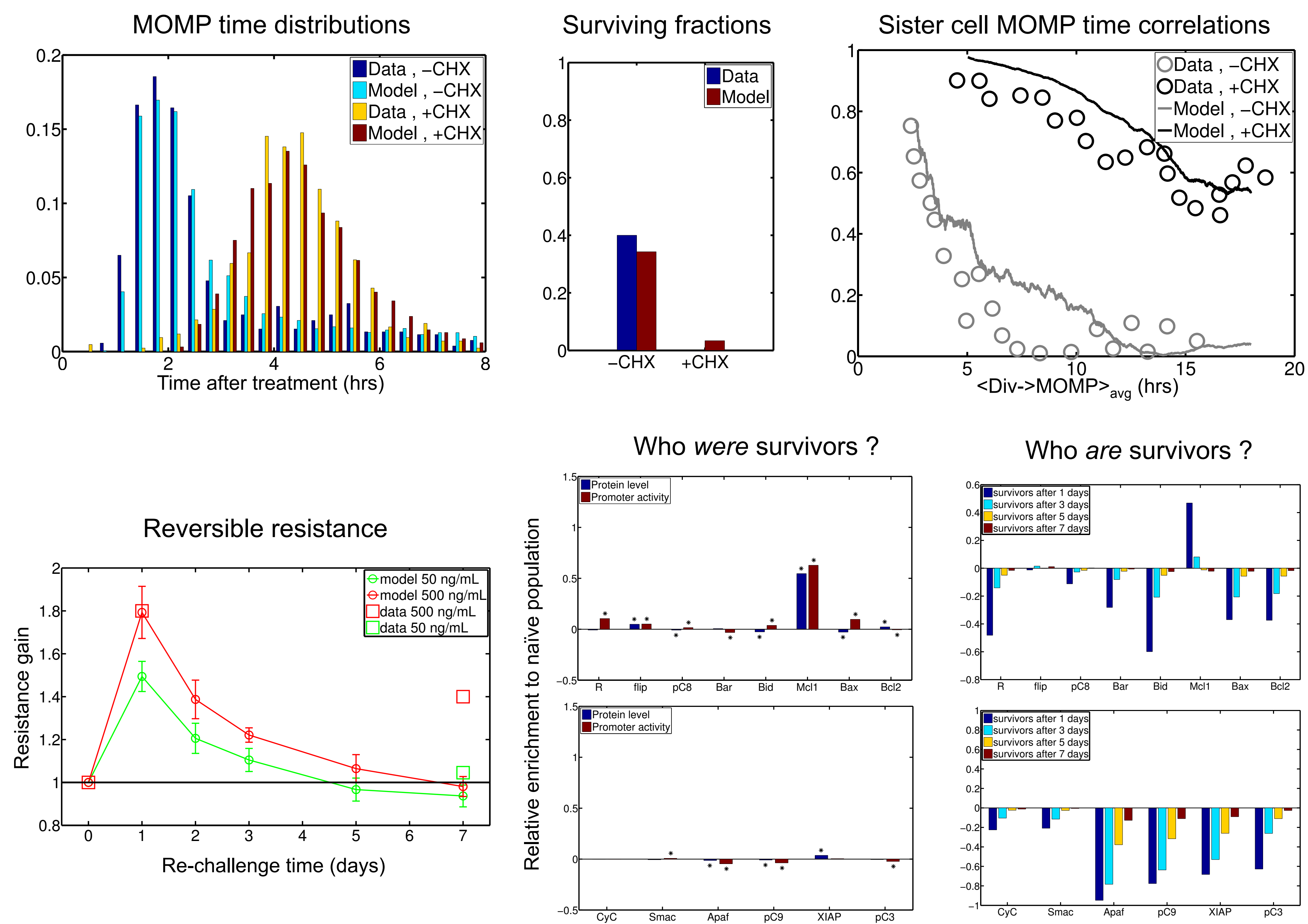
MOMP time distributions
Data , −CHX
Model , −CHX
Data , +CHX
Model , +CHX
Time after treatment (hrs)
Surviving fractions
Data
Model
−CHX
+CHX
Sister cell MOMP time correlations
Data , −CHX
Data , +CHX
Model , −CHX
Model , +CHX
<Div->MOMP>avg (hrs)
Reversible resistance
model 50 ng/mL
model 500 ng/mL
data 500 ng/mL
data 50 ng/mL
Resistance gain
Re-challenge time (days)
Who were survivors ?
Protein level
Promoter activity
R
flip
pC8
Bar
Bid
Mcl1
Bax
Bcl2
CyC
Smac
Apaf
pC9
XIAP
pC3
Relative enrichment to naive population
Who are survivors ?
survivors after 1 days
survivors after 3 days
survivors after 5 days
survivors after 7 days
