# Supplementary material for: Modeling Dynamics of Cell-to-Cell Variability in TRAIL-Induced Apoptosis Explains Fractional Killing and Predicts Reversible Resistance
Source: PLoS Comput Biol. 2014 Oct 23;10(10):e1003893. doi: 10.1371/journal.pcbi.1003893 (PMC4207462; doi:10.1371/journal.pcbi.1003893)
Supplement: Figure S10 — Robustness of short-term model behavior regarding the presence/absence of feedback loop and the degradation of active forms. Model-Data agreement is shown for MOMP time distributions, surviving fractions and sisters correlation of MOMP time in both treatment conditions for model variants when the C3->C6->C8 feedback loop is either present/absent and the default active forms half-life is 27, 15, 5 or 2 hours. Significant model-data deviation is seen only for the fastest active forms degradation. (PDF) [file pcbi.1003893.s010.pdf]

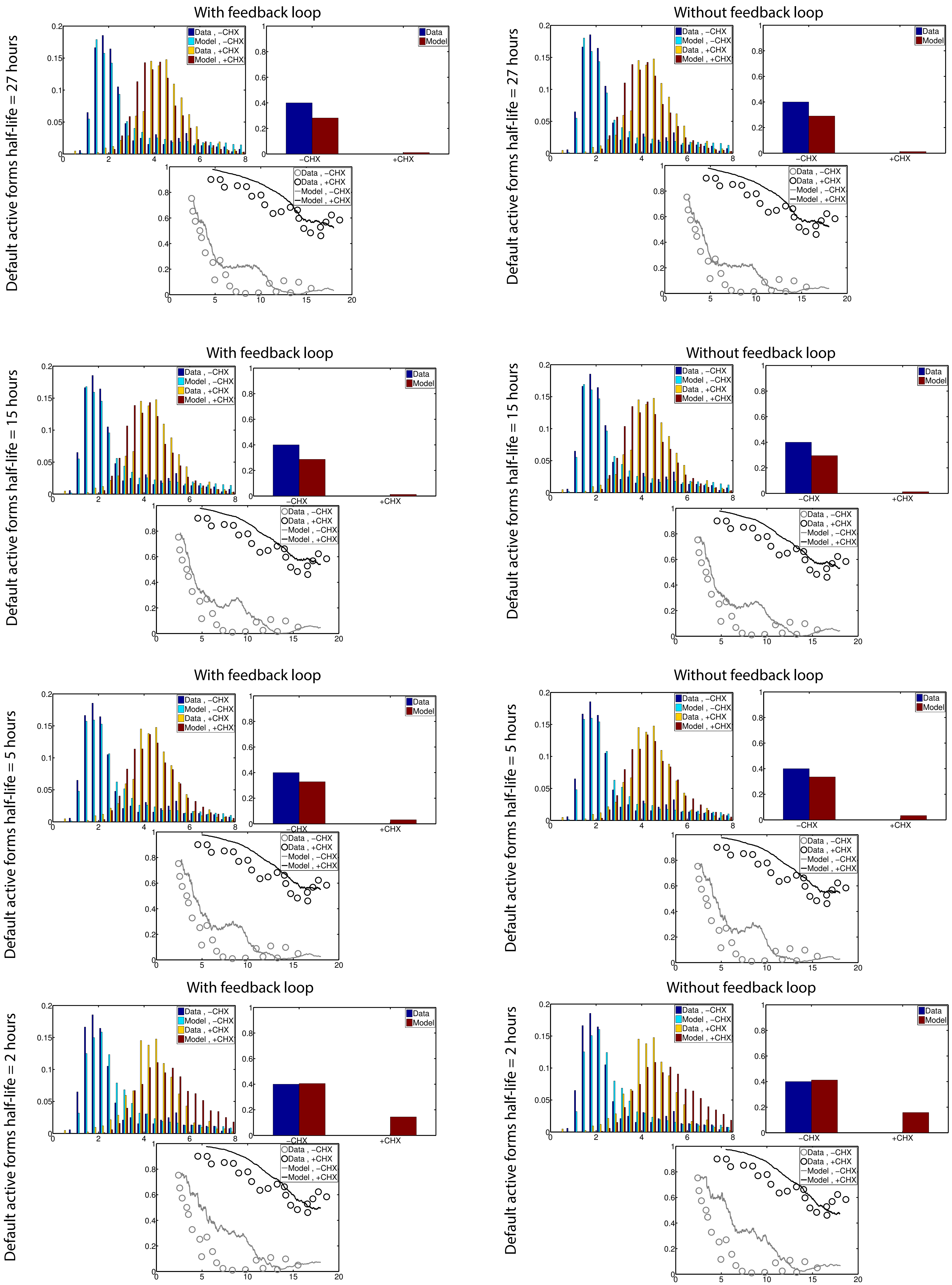

With feedback loop
Without feedback loop
Default active forms half-life = 27 hours
Default active forms half-life = 15 hours
Default active forms half-life = 5 hours
Default active forms half-life = 2 hours
Data , −CHX
Model , −CHX
Data , +CHX
Model , +CHX
Data
Model
−CHX
+CHX
