# Supplementary material for: Modeling Dynamics of Cell-to-Cell Variability in TRAIL-Induced Apoptosis Explains Fractional Killing and Predicts Reversible Resistance
Source: PLoS Comput Biol. 2014 Oct 23;10(10):e1003893. doi: 10.1371/journal.pcbi.1003893 (PMC4207462; doi:10.1371/journal.pcbi.1003893)
Supplement: Table S1 — Standard stochastic protein turnover models. (DOCX) [file pcbi.1003893.s013.docx]

**Table S1. Standard stochastic protein turnover models**

|  | Constraints | | | | Rate values | | | | | |
| --- | --- | --- | --- | --- | --- | --- | --- | --- | --- | --- |
| Protein | Switching speed k_on_+k_off_  (hrs^-1^)  [36]  *fixed* | Mean mRNA level  [sup. ref. 6, see Text S1]  *fixed* | Mean protein  level  [13]  *fixed* | Protein level Coefficient of Variation  [13]  *fixed* | Off to on promoter rate  k_on_  (hrs^-1^)  *derived* | On to off promoter rate  k_off_  (hrs^-1^)  *derived* | k_sm_  (min^-1^)  *derived* | mRNA deg. rate  γ_m_  (hrs^-1^)  *fixed* | Protein synth. rate  k_sp_  (min^-1^)  *derived* | Protein deg. rate  γ_p_  (hrs^-1^)  *fixed* |
| Receptor | 10.44 | 17 | 1000 | 0.25 | 0.388 | 10.05 | 0.587 | 0.077 | 0.025 | 0.0257 |
| Caspase-8 |  |  | 10000 | 0.25 | 0.388 | 10.05 | 0.587 |  | 0.25 |  |
| Bar |  |  | 1000 | 0.25 | 0.388 | 10.05 | 0.587 |  | 0.025 |  |
| Caspase-3 |  |  | 10000 | 0.282 | 0.289 | 10.15 | 0.789 |  | 0.25 |  |
| Caspase-6 |  |  | 10000 | 0.25 | 0.388 | 10.05 | 0.587 |  | 0.25 |  |
| XIAP |  |  | 100000 | 0.288 | 0.275 | 10.17 | 0.829 |  | 2.52 |  |
| PARP |  |  | 1000000 | 0.25 | 0.388 | 10.05 | 0.587 |  | 25.2 |  |
| Bid |  |  | 60000 | 0.288 | 0.275 | 10.17 | 0.829 |  | 1.51 |  |
| Bax |  |  | 80000 | 0.271 | 0.318 | 10.12 | 0.717 |  | 2.01 |  |
| Bcl-2 |  |  | 30000 | 0.294 | 0.268 | 10.18 | 0.871 |  | 0.76 |  |
| Pore |  |  | 500000 | 0.25 | 0.388 | 10.05 | 0.587 |  | 12.6 |  |
| CytoC_m |  |  | 500000 | 0.25 | 0.388 | 10.05 | 0.587 |  | 12.6 |  |
| Smac |  |  | 100000 | 0.25 | 0.388 | 10.05 | 0.587 |  | 2.51 |  |
| Apaf |  |  | 100000 | 0.25 | 0.388 | 10.05 | 0.587 |  | 2.51 |  |
| Caspase-9 |  |  | 100000 | 0.25 | 0.388 | 10.05 | 0.587 |  | 2.51 |  |
