# Supplementary material for: Modeling Dynamics of Cell-to-Cell Variability in TRAIL-Induced Apoptosis Explains Fractional Killing and Predicts Reversible Resistance
Source: PLoS Comput Biol. 2014 Oct 23;10(10):e1003893. doi: 10.1371/journal.pcbi.1003893 (PMC4207462; doi:10.1371/journal.pcbi.1003893)
Supplement: Table S2 — Specific stochastic protein turnover models. (DOCX) [file pcbi.1003893.s014.docx]

**Table S2. Specific stochastic protein turnover models**

“Non-fitted” model (Fig. S6):

|  | Constraints | | Rate values | | | | | |
| --- | --- | --- | --- | --- | --- | --- | --- | --- |
| Protein | Mean mRNA level  [sup. ref. 6, see Text S1] | Mean protein level  [13] | k_on_ (hrs^-1^) | k_off_ (hrs^-1^) | k_sm_ (min^-1^) | γ_m_ (hrs^-1^) | k_sp_ (min^-1^) | γ_p_ (hrs^-1^) |
| Flip | 17 | 2000 | 0.388 | 10.05 | 2.6186 | 0.3466 | 2.7182 | 1.39 |
| Mcl-1 | 17 | 20000 | 0.388 | 10.05 | 2.6186 | 0.3466 | 27.182 | 1.39 |

“Fitted” model (Figs. 3,4,6,7):

|  | Constraints | | Rate values | | | | | |
| --- | --- | --- | --- | --- | --- | --- | --- | --- |
| Protein | Mean mRNA level  [sup. ref. 6, see Text S1] | Mean protein level  [13] | k_on_ (hrs^-1^) | k_off_ (hrs^-1^) | k_sm_ (min^-1^) | γ_m_ (hrs^-1^) | k_sp_ (min^-1^) | γ_p_ (hrs^-1^) |
| Flip | 17 | 2000 | 0.0417 | 0.0625 | 0.4910 | 0.6931 | 3.3978 | 1.73 |
| Mcl-1 | 17 | 20000 | 0.0417 | 0.0625 | 0.4910 | 0.6931 | 33.9780 | 1.73 |
