# Supplementary material for: Modeling Dynamics of Cell-to-Cell Variability in TRAIL-Induced Apoptosis Explains Fractional Killing and Predicts Reversible Resistance
Source: PLoS Comput Biol. 2014 Oct 23;10(10):e1003893. doi: 10.1371/journal.pcbi.1003893 (PMC4207462; doi:10.1371/journal.pcbi.1003893)
Supplement: Table S3 — Non-native form degradation. (DOCX) [file pcbi.1003893.s015.docx]

**Table S3. Non-native form degradation**

| Protein | Half-life (hours) | Reference |
| --- | --- | --- |
| TRAIL | 9 | [sup. ref. 9, see Text S1] |
| Pore* | 1.9 | [13] |
| Flip:Receptor | same as Flip |  |
| Mcl-1:tBid | same as Mcl-1 |  |
| All others | 5 | [sup. refs. 10-14, see Text S1] |
