# Supplementary material for: Modeling Dynamics of Cell-to-Cell Variability in TRAIL-Induced Apoptosis Explains Fractional Killing and Predicts Reversible Resistance
Source: PLoS Comput Biol. 2014 Oct 23;10(10):e1003893. doi: 10.1371/journal.pcbi.1003893 (PMC4207462; doi:10.1371/journal.pcbi.1003893)
Supplement: Text S1 — Supplementary results and supplementary methods. (DOCX) [file pcbi.1003893.s016.docx]

# Text S1: Supplementary Information

# *Modeling dynamics of cell-to-cell variability in TRAIL-induced apoptosis explains* *fractional killing and reversible resistance*

François Bertaux, Szymon Stoma, Dirk Drasdo, Gregory Batt

- **Supplementary Results**
- Biochemistry of TRAIL-induced apoptosis
- Modeling stochastic protein turnover
- Short-lived protein fluctuations are more sensitive to bursting kinetics
- Modeling stochastic protein turnover in TRAIL-induced apoptosis
- Stochastic protein turnover models predicts transient memory in cell sensitivity to TRAIL and CHX
- Behavior of the “non-fitted” model
- Mcl1 and Flip fluctuations for the “non-fitted” and “fitted” model
- Large, rare fluctuations of Mcl1 alone is sufficient to explain all observations
- Representative single-cell trajectories before and after TRAIL treatments
- Influence of C3->C6->C8 feedback loop and of non-native forms degradation
- **Supplementary Methods**
- Simulating TRAIL-induced apoptosis with stochastic protein turnover
- In-silico repeated TRAIL experiment
- In-silico sister cells experiment
- Quantification of model/data agreement
- **Supplementary References**

**Supplementary Results**

### Biochemistry of TRAIL-induced apoptosis

The activation of the death receptor DR4/5 by TRAIL binding promotes the assembly of the death-inducing signaling complexes (DISC), which recruit and activate initiator caspases like caspase-8 (C8) [sup. ref. 1]. Once activated, initiator caspases cleave and activate effector caspases such as caspase-3 (C3). Effector caspases cleave essential structural proteins, inhibitors of DNase, and DNA repair proteins (PARP), eventually leading to cell death. The cellular effect of effector caspase activation is regulated by factors such as XIAP, which blocks the proteolytic activity of caspase-3 by binding tightly to its active site and promotes its degradation via ubiquitination [sup. ref. 2]. In addition to the direct activation of effector caspases, initiator caspases also activate Bid and Bax [sup. ref. 3]. If not kept in check by inhibitors, most notably Bcl2, activated Bax directly contributes to the formation of pores in the mitochondria outer membrane, leading to MOMP [sup. ref. 4]. Following MOMP, critical apoptosis regulators, such as Smac and cytochrome c (CyC), translocate into the cytoplasm. Smac binds to and inactivates XIAP, thus relieving the inhibition of effector caspases by XIAP [sup. ref. 5]. Cytochrome c combines with Apaf-1 to form the apoptosome that in turn activates the initiator caspase-9 (C9) that activates effector caspases. A simplified view is given in Fig. S1.

### Modeling stochastic protein turnover

We model protein turnover with a stochastic process describing mRNA level fluctuations (promoter activity switches, mRNA production and degradation are stochastic events), and deterministic processes for protein translation and degradation (Fig. S2).

To constrain model rates based on experimentally available information, one can use analytical expressions characterizing steady-state mRNA and protein level distributions (moments) and their fluctuations (autocorrelations functions). The derivation of steady-state distribution moments can be done by performing Laplace transforms (equivalently referred as generating functions) on the steady-state formulation of the chemical master equation. The obtained system of equations is closed because there are no reactions of order 2 or higher. It has been done by Paszek [39] and yields for example the following moments for the steady-state distribution:

- Promoter activity (denoted by , equal to either 0 or 1)

- mRNA level (denoted by , taking discrete values)
- Protein level (denoted by , taking continuous values)

We now present the derivation of the autocorrelation function. The first step is to obtain the differential equations describing the temporal evolution of the first moments. Such equations are obtained by using the master equation, which gives how the joint probability distribution is changed during an infinitesimal time interval because of the reactions that can occur:

For example, if one is interested in the temporal evolution of

Noticing that , and that the probability distribution cannot charge states where or , one gets:

With similar calculations, one also obtains the two following equations:

This system of autonomous ordinary differential equations is linear and can be solved analytically. In particular, we have:

where:

This particular ordering has been chosen to facilitate the next step of the auto-correlation function derivation, which is to find an appropriate expression for the quantity :

letting the expectation of when starting from a dirac in at appear:

letting crossed second order moments of the steady-state distribution appear:

We thus have:

and therefore:

Finally, after having plugged the expressions for the covariances (given in [39]) and for the functions and , we get a final expression (i.e. containing only terms for which expression in terms of rate parameters has been given already) for the autocorrelation function:

with:

The exactness of this expression was verified by Monte-Carlo simulations using Gillespie algorithm, for a parameter set carefully chosen such that each term in the expression has a non-negligible contribution to the function value. One can note how the three time-scales , corresponding to the promoter, the mRNA and the protein respectively, are convoluted together in this expression.

### Short-lived protein fluctuations are more sensitive to bursting kinetics

Intuitively, fluctuations of short-lived proteins are expected to be more sensitive to the precise kinetics of bursting than long-lived proteins. Using protein level variability and half-autocorrelation time to characterize protein fluctuations, this hypothesis was confirmed from the corresponding analytical expressions (Fig. S3). This motivated us to consider standard stochastic protein turnover models for long-lived proteins but to give particular attention to the few short-lived proteins.

### Modeling stochastic protein turnover in TRAIL-induced apoptosis

We apply our approach to TRAIL-induced apoptosis. We use EARM kinetic model [5,13] to describe protein-protein reactions taking place between TRAIL death exposure and cell death commitment. It comprises 17 native proteins and 41 other species involved in 71 reactions.

Unless required, we equipped all native proteins with the same default model of stochastic protein turnover. We used median values for mRNA levels, protein and mRNA half-lives from measured distributions in mammalian cells [sup.ref. 6]. At the promoter level, switching rates were estimated for a dozen of genes [36], and we used measured values to constrain model reaction rates. Remaining rates were deduced from the mean and variance of protein level present as initial condition in [13] using analytical expressions derived from the stochastic protein turnover model (Fig. S4, Table S1).

As mentioned earlier, short-lived proteins should be given particular attention: transcriptional bursts are smoothed out in the fluctuations of a stable protein while fully revealed when both mRNA and protein exhibit fast turnover. Flip and Mcl1 are known to exhibit very fast turnover [42,43,44]. Measurements in mouse ES cells [sup. ref. 7] also suggest that Flip and Mcl1 transcripts are particularly short-lived. Therefore, we considered a specific stochastic protein turnover model for those two proteins, exploring realistic ranges for promoter switching rates, mRNA half-life and protein half-life (Fig. S4).

Non-native protein species (catalytically activated forms or complexes) are also subjected to degradation. We used a half-life of 9 hours for TRAIL (as measured in [sup. ref. 8]), 1.9 hours for the mitochondrial pores (as in [13]) and the half-life of the native form for complexes involving Flip or Mcl1. For all other species we used a unique value (5 hours) to account for the fact that active forms are usually degraded faster sup. refs. [9]-[13] values recapitulated in Table S3, see dedicated later section for discussion on the influence of this choice). We also assumed the feedback loop C3->C6->C8 to be absent (authors comment to [5], on editor's website). Otherwise, important cell death was still seen days after TRAIL treatment (see dedicated section for discussion on the feedback loop influence). It has been observed that protein synthesis is substantially decreased during apoptosis [sup. ref. 14]. Because this effect seems to happen essentially after effector caspases activation, when cells are already committed to death, we did not include it in our model.

### Stochastic protein turnover models predicts transient memory in cell sensitivity to TRAIL and CHX

See Fig. S5.

### The “non-fitted” model quantitatively predicts TRAIL+CHX single-cell data and lead to fractional killing and reversible resistance for TRAIL alone treatments

See Fig. S6.

### Mcl1 and Flip fluctuations for the “non-fitted” and “fitted” model

See Fig. S7.

**Large, rare fluctuations of Mcl1 alone are sufficient to explain cell fate variability and transient inheritance in both conditions**

See Fig. S8.

### Representative single-cell trajectories before and after TRAIL treatments

See Fig. S9.

### Influence of C3->C6->C8 feedback loop and of non-native forms degradation

In presence of the feedback loop (as in the original model from Spencer et al., [13]) or with low degradation rates for non-native forms (also as in the original model), model predicts an extinction (or a non-reconstruction) of the cell number at 7 days after the treatment, in contradictions with observations.

Because the functional role of this feedback loop is debated (authors comment to [5], on editor’s website) and non-native forms are often subjected to active degradation sup. refs. [9]-[13], we investigate whether realistic changes could re-conciliate the model with experimental observations.

We tested the effect of the presence/absence of the feedback loop as well as the influence of the value of the default non-native forms degradation rate (varied in a broad interval, between 2 to 27 hours in half-life). As shown in Figs. S10, S11 and S12, model predictions are remarkably robust, with the exception of population reconstruction after 7 days. This criterion led us, in the model, to assume that the feedback loop is absent and to use 5 hours as the default half-life of non-native forms.

## Supplementary Methods

### Simulating TRAIL-induced apoptosis with stochastic protein turnover

In general, the TRAIL signaling protein-protein reactions are taking place concurrently with stochastic protein turnover. When the noise in signaling reactions is neglected due to high protein copy number, those reactions can be simulated using ODEs. However, rates of protein synthesis are in our model stochastic, as they follow mRNA fluctuations.

Promoter activity and mRNA fluctuations were simulated using an implementation of the Gillespie algorithm in C++ (Numerical Recipes). Messenger RNA trajectories were computed and stored in advance because protein levels do not affect the rates of promoter state switches, mRNA production and degradation. The ODEs governing evolution of all protein levels were then simulated using the Semi-Implicit Extrapolation method implemented in C++ ([sup. ref. 15], Numerical Recipes). This method was significantly faster than a more standard Runge-Kutta method (Dormand-Prince, C++, Numerical Recipes) but gave identical results.

### In-silico sister cells experiment

To sample the state (promoter activity, mRNA and protein levels) of the mother cells, all stochastic protein turnover models were simulated during 25 days (Monte-Carlo sampling) for each of the 10^4^ (10^5^ for results presented in main text) mother cells. This duration was verified by comparison with analytical results to be sufficient to reach the steady-state distribution. Sister cells were simply constructed by duplication of the mother cell state.

Because in experiments from [13], the distribution of durations between division and treatment was not uniform (see Figs. S5-b and 1-g in [13]), we applied a sampling algorithm to approximately reproduce those distributions. The overall impact on correlation curves was generally low compared to results obtained with assuming a fully uniform distribution of division time in the pre-stimulus recording interval. MOMP was considered to have occurred when half of mitochondrial Smac has been released.

### In-silico repeated TRAIL experiment

A naïve population of 10^4^ cells was obtained as in the sister cells experiment. Each cell was assigned a random time of next division. To account for the fact that the distribution of next division times is not uniform in growing cell populations, we used a distribution obtained by simulating simple growth. New cells were attributed a next division time according to a cell cycle duration normally distributed with 27 hours mean and 3 hours standard deviation. Cells in which cPARP levels exceeded 10^5^ were considered dead as in [25]. To closely mimic the experimental protocol used in [14], we accounted for the effect of passing cells by checking population size each day and if needed, removing randomly cells until 10^4^ were left. Resistance gain is computed as where , similarly to [14].

### Quantification of model-data agreement

For the estimation Flip/Mcl1 model rates based cell fate variability experimental data, for the validation against transient cell fate inheritance data and for robustness analysis (Figs. 5A-C, 5E), it is needed to quantify the agreement/discrepancy between each model tested and the observed data. Such quantification was performed as follows:

- MOMP time distribution

Data was extracted from [13] (Fig. S4 b and c). It consists in MOMP time histograms (number of cells which did MOMP in a given 20 minutes time interval between 0 and 8 hours after treatment, 24 intervals in total). It was transformed in MOMP time frequencies by dividing by the total cell number. The same MOMP time frequencies were computed from simulated results. An agreement cost was then computed as the squared deviation between the two sets of frequencies, which respectively represent the empirical/model MOMP time distributions.

- Surviving fraction

An agreement cost for surviving fractions was simply computed as the squared difference between the surviving fractions observed experimentally and in simulations 8 hours after treatment.

- Sister cell MOMP time correlation curve

Spencer et al. [13] quantified the transient inheritance of MOMP times by computing a curve of sister cells MOMP time correlation as follows: pairs of sister cells for which both cells did MOMP before 8 hours were sorted as a function of the average time between division and MOMP, and linear regression correlation coefficients were computed for all groups obtained by sliding a window of constant size along the sorted pairs. For each group, mean time between division and MOMP was also computed, thus providing the abscissa of the corresponding point in the curve. From this data (Figs. S5-d in [13]), twenty representative points were extracted. To compute a comparable curve from simulations results, we applied the same quantification of sister cell MOMP time correlations. The group size was chosen such that fraction of total pairs in each group is 10%, similarly to what has been done in [13]. One should not that the correlation values are available at different time points between the experimental and simulated curves. Thus, to permit a quantification of the agreement cost, each point in the experimental curve was mapped to the point in the simulated curve for which time points are the closest. The cost then penalizes, for each pair of points, a difference in the correlations but also in the time. In a formal manner, if for each point in the data curve, the point in the simulated curve such that is the closest to , the cost is then computed as:

- Comparison between the different types of data

To permit comparison between the different types of data, in each case a threshold for the cost defining agreement/disagreement was manually set by visual comparison of experimental and simulated data. For visualization purposes, Fig. 5-A,B,E represent a linearly normalized cost such that the threshold value correspond to 0.5 (represented in yellow). Normalized costs above 1 are capped to 1 and represented in red. The threshold costs used were 0.01/0.01, 0.01/0.01, and 2/2.5 for MOMP time distributions, surviving fractions and sister cell correlations respectively (TRAIL+CHX condition/TRAIL alone condition). To assign an agreement cost for MOMP time distribution AND surviving fractions (Fig. 5C), the maximum of the two normalized costs is taken.

## Supplementary References

[1] F. C. Kischkel, S. Hellbardt, I. Behrmann, M. Germer, M. Pawlita, P. H. Krammer, and M. E. Peter, “Cytotoxicity-dependent APO-1 (Fas/CD95)-associated proteins form a death-inducing signaling complex (DISC) with the receptor.,” *EMBO J.*, vol. 14, no. 22, pp. 5579–5588, Nov. 1995.

[2] Q. L. Deveraux, N. Roy, H. R. Stennicke, T. Van Arsdale, Q. Zhou, S. M. Srinivasula, E. S. Alnemri, G. S. Salvesen, and J. C. Reed, “IAPs block apoptotic events induced by caspase-8 and cytochrome c by direct inhibition of distinct caspases.,” *EMBO J.*, vol. 17, no. 8, pp. 2215–2223, Apr. 1998.

[3] X. Luo, I. Budihardjo, H. Zou, C. Slaughter, and X. Wang, “Bid, a Bcl2 interacting protein, mediates cytochrome c release from mitochondria in response to activation of cell surface death receptors,” *Cell*, vol. 94, no. 4, pp. 481–490, 1998.

[4] H. Kim, M. Rafiuddin-Shah, H.-C. Tu, J. R. Jeffers, G. P. Zambetti, J. J.-D. Hsieh, and E. H.-Y. Cheng, “Hierarchical regulation of mitochondrion-dependent apoptosis by BCL-2 subfamilies.,” *Nat. Cell Biol.*, vol. 8, no. 12, pp. 1348–1358, Dec. 2006.

[5] C. Du, M. Fang, Y. Li, L. Li, and X. Wang, “Smac, a mitochondrial protein that promotes cytochrome c-dependent caspase activation by eliminating IAP inhibition.,” *Cell*, vol. 102, no. 1, pp. 33–42, Jul. 2000.

[6] B. Schwanhäusser, D. Busse, N. Li, G. Dittmar, J. Schuchhardt, J. Wolf, W. Chen, and M. Selbach, “Global quantification of mammalian gene expression control,” *Nature*, vol. 473, no. 7347, pp. 337–342, May 2011.

[7] L. V. Sharova, A. A. Sharov, T. Nedorezov, Y. Piao, N. Shaik, and M. S. H. Ko, “Database for mRNA Half-Life of 19 977 Genes Obtained by DNA Microarray Analysis of Pluripotent and Differentiating Mouse Embryonic Stem Cells,” *DNA Research*, vol. 16, no. 1, pp. 45–58, Jan. 2009.

[8] Y. S. Youn, M. J. Shin, S. Y. Chae, C.-H. Jin, T. H. Kim, and K. C. Lee, “Biological and physicochemical evaluation of the conformational stability of tumor necrosis factor-related apoptosis-inducing ligand (TRAIL),” *Biotechnol Lett*, vol. 29, no. 5, pp. 713–721, Feb. 2007.

[9] K. Breitschopf, “Ubiquitin-mediated Degradation of the Proapoptotic Active Form of Bid. A Functional Consequence on Apoptosis Induction,” *Journal of Biological Chemistry*, vol. 275, no. 28, pp. 21648–21652, May 2000.

[10] E. Ferraro, A. Pulicati, M. T. Cencioni, M. Cozzolino, F. Navoni, S. di Martino, R. Nardacci, M. T. Carrì, and F. Cecconi, “Apoptosome-deficient cells lose cytochrome c through proteasomal degradation but survive by autophagy-dependent glycolysis.,” *Mol. Biol. Cell*, vol. 19, no. 8, pp. 3576–3588, Aug. 2008.

[11] B. Li and Q. P. Dou, “Bax degradation by the ubiquitin/proteasome-dependent pathway: involvement in tumor survival and progression.,” *Proc. Natl. Acad. Sci. U.S.A.*, vol. 97, no. 8, pp. 3850–3855, Apr. 2000.

[12] P. Tawa, K. Hell, A. Giroux, E. Grimm, Y. Han, D. W. Nicholson, and S. Xanthoudakis, “Catalytic activity of caspase-3 is required for its degradation: stabilization of the active complex by synthetic inhibitors.,” *Cell Death Differ.*, vol. 11, no. 4, pp. 439–447, Apr. 2004.

[13] J. A. Thorpe, P. A. Christian, and S. R. Schwarze, “Proteasome inhibition blocks caspase-8 degradation and sensitizes prostate cancer cells to death receptor-mediated apoptosis,” *Prostate*, vol. 68, no. 2, pp. 200–209, 2007.

[14] M. Bushell, M. Stoneley, Y. W. Kong, T. L. Hamilton, K. A. Spriggs, H. C. Dobbyn, X. Qin, P. Sarnow, and A. E. Willis, “Polypyrimidine tract binding protein regulates IRES-mediated gene expression during apoptosis.,” *Molecular Cell*, vol. 23, no. 3, pp. 401–412, Aug. 2006.

[15] P. Deuflhard, “Recent Progress in Extrapolation Methods for Ordinary Differential Equations,” *SIAM Rev.*, vol. 27, no. 4, pp. 505–535, Dec. 1985.
